# OptoPi: An open source flexible platform for the analysis of small animal behaviour

**DOI:** 10.1016/j.ohx.2023.e00443

**Published:** 2023-06-19

**Authors:** Xavier Cano-Ferrer, Ruairí J.V. Roberts, Alice S. French, Joost de Folter, Hui Gong, Luke Nightingale, Amy Strange, Albane Imbert, Lucia L. Prieto-Godino

**Affiliations:** The Francis Crick Institute, London NW1 1BF, United Kingdom

**Keywords:** Behaviour, Open hardware, 3D-printing, Laser cutting, Optogenetics, Light stimulation, LED, Arduino, Neuroscience

## Abstract

Behaviour is the ultimate output of neural circuit computations, and therefore its analysis is a cornerstone of neuroscience research. However, every animal and experimental paradigm requires different illumination conditions to capture and, in some cases, manipulate specific behavioural features. This means that researchers often develop, from scratch, their own solutions and experimental set-ups. Here, we present OptoPi, an open source, affordable (∼ £600), behavioural arena with accompanying multi-animal tracking software. The system features highly customisable and reproducible visible and infrared illumination and allows for optogenetic stimulation. OptoPi acquires images using a Raspberry Pi camera, features motorised LED-based illumination, Arduino control, as well as irradiance monitoring to fine-tune illumination conditions with real time feedback. Our open-source software (BioImageProcessing) can be used to simultaneously track multiple unmarked animals both in on-line and off-line modes. We demonstrate the functionality of OptoPi by recording and tracking under different illumination conditions the spontaneous behaviour of larval zebrafish as well as adult *Drosophila* flies and their first instar larvae*,* an experimental animal that due to its small size and transparency has classically been hard to track. Further, we showcase OptoPi’s optogenetic capabilities through a series of experiments using transgenic *Drosophila* larvae.

## Graphical abstract

**Figure:**
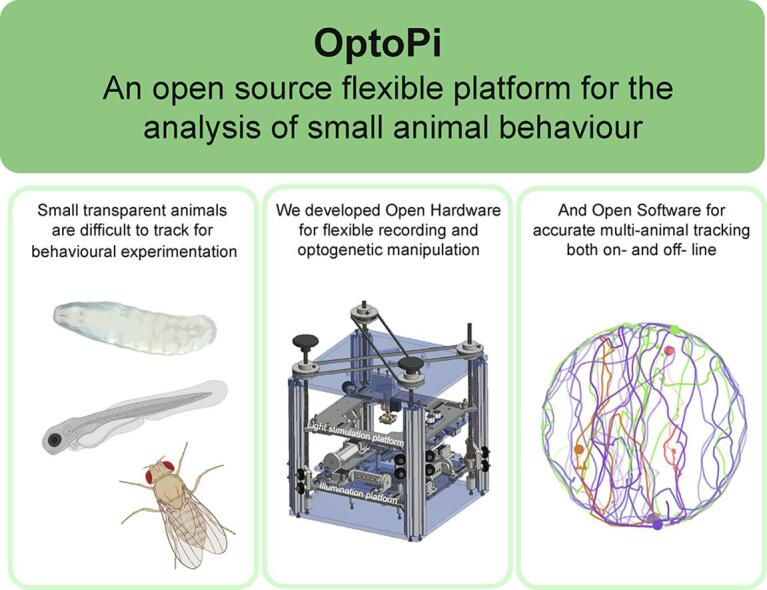

## Specifications table

**Table:** 

Hardware name	OptoPi
Subject area	Neuroscience
Hardware type	Animal BehaviourLight stimulationImaging tools
Open Source License	GNU General Public License v3.0
Cost of Hardware	∼ £600
Source File Repository	https://doi.org/10.17605/OSF.IO/PU8F5*https://github.com/PrietoGodinoLab/OptoPi*

## 1 Hardware in context

A key method in neuroscience is behavioural analysis, including the manipulation of neuronal activity while monitoring behavioural output. A commonly used approach to accurately modify neuronal activity is optogenetics, whereby animals are genetically engineered to express light-sensitive ion channels in specific neuronal populations. This method is particularly accessible for small animals such as *Drosophila* and zebrafish where stimulation can be delivered in freely behaving individuals.

Over the last decade numerous open hardware solutions have been published to facilitate the study of small animal behaviour. These range from relatively generic and modular systems such as the FlyPi [1], able to record and optogenetically stimulate animals, to more sophisticated and task-specific set-ups. For example, Ethoscopes were designed specifically to measure and manipulate the sleep of flies in real time through close-loop experiments, but can also be adapted for other assays [2]. Another excellent example is PiVR the first open source behavioural device designed to perform closed loop experiments with light stimulation [3]. However, a limitation of these previous designs is that although their performance is excellent for the specific task for which they were developed, adapting them to different purposes, such as using different experimental animals, often requires modifications of the illumination and/or stimulation conditions, some of which can be difficult or time-consuming to implement.

To overcome these limitations, here we present a hardware solution that enables the experimenter to change the illumination, recording, and optogenetic stimulation conditions flexibly and rapidly. Our platform consists of a stage to place the animal arena and multiple movable illumination modules whose angle, light intensity and wavelength can be adjusted in a matter of seconds with user-friendly knobs. Additionally, the camera distance to the arena can also be easily modified. Together these adaptations enable rapid control over recording conditions to find settings that are optimal for each experiment. Our device is also fitted with multiple wavelength LEDs that match the activation spectrum of the most popularly used optogenetic actuators. These are placed onto adjustable modules to ensure optimal optogenetic stimulation. To quickly reproduce previous recording and stimulation settings, OptoPi is equipped with spectrum, irradiance and ultrasound sensors that monitor illumination conditions and placement of illumination modules. Finally, we also describe the development of a new open-source tracking software, which can be used in on and off-line modes, and that enables the tracking of multiple animals while keeping individual identity after collisions.

We anticipate that the flexibility of our platform will enable researchers to have a single solution for multiple behavioural analysis needs within their lab and will help the standardisation of behavioural assays in small animals. In addition, OptoPi’s hardware could be combined with previously published modules to expand its functionality. For example, the camera recording settings could be controlled using the graphical using interface (GUI) of the FlyPi, or our hardware could be combined with the software capabilities of PiVR for close-loop experiments. This cross-platform compatibility is enabled by the fact that all these low-cost platforms are based on the same basic hardware, i.e. Rapsberry Pi camera and out of the self LED arrays. Therefore, OptoPi expands the open source toolkit that neuroscience researchers can take advantage of to perform behavioural analyses in a wide variety of animals and settings.

## 2 Hardware description.

This section has the following subsections:

2.1. Overview.

2.2. Illumination control.

2.3. Optogenetic modules.

2.4. Recording settings.

2.5. Tracking options.

### 2.1 Overview

**Fig. 1:**
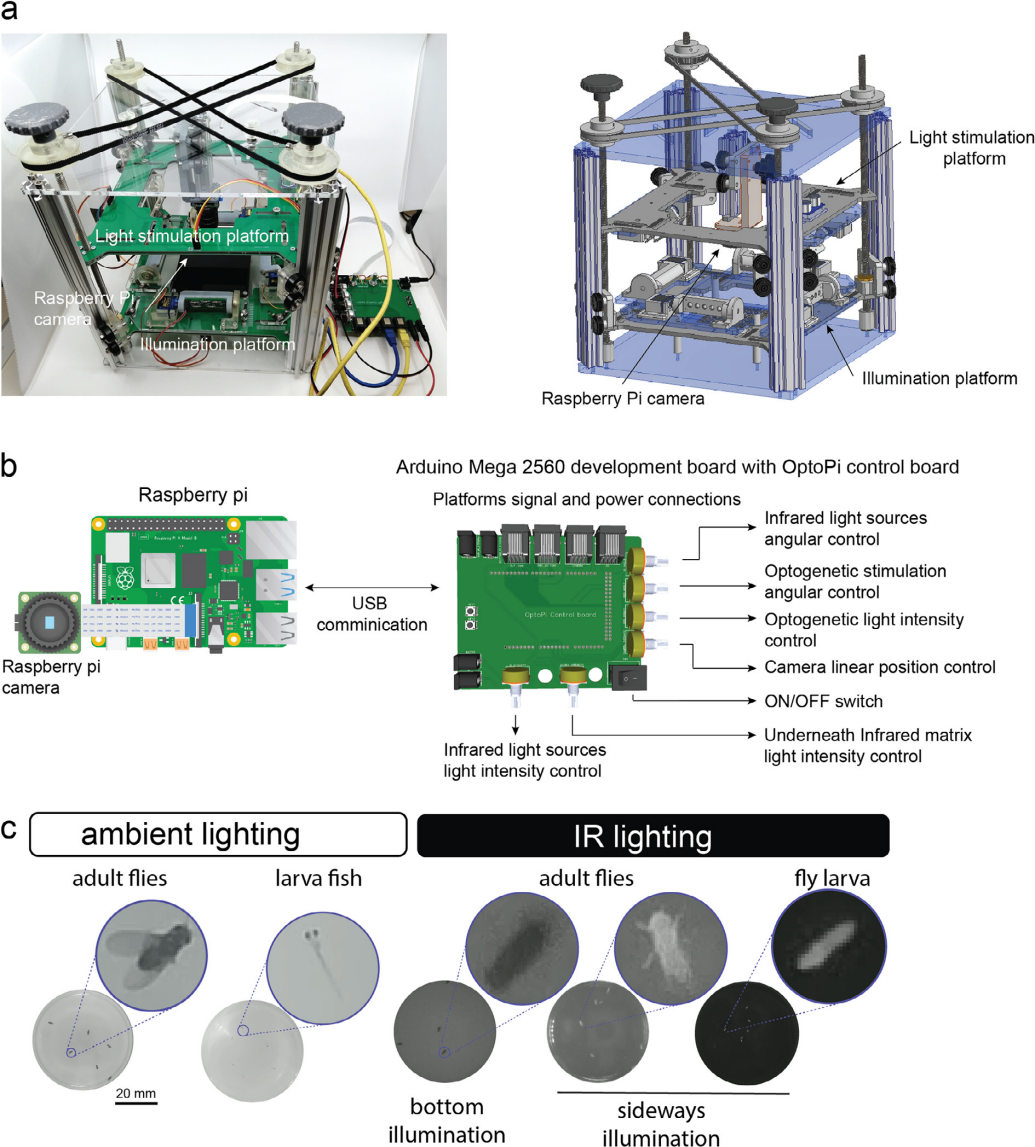
Fly arena design. a. Mechanical assembly of OptoPi using CAD software on the right. The stimulation and illumination platforms that surround the arena move freely and independently. Fully assembled arena on the left with the Raspberry Pi HQ camera visible in the centre. b. The Raspberry Pi both controls the camera, which records the experiment, and communicates via serial port with the Arduino Mega 2560. The Arduino Mega 2560 has its own shield to allow the researcher to control the source, position and intensity of light as well as the camera position. c. Examples of different lighting configurations. Ambient lighting works best for dark animals such as adult flies and larvae zebrafish when no optogenetic manipulation is required. For optogenetic manipulation and transparent animals such as fly larvae, IR lighting is preferred.

OptoPi consists of a mechanical framework, composed of 3D printed, laser-cut and mechanical parts such as the four metal posts (Fig. 1a). The behavioural arena is placed on a static platform, that can have its height adjusted, while the illumination, stimulation and camera modules are movable to achieve optimal experimental conditions. The device is controlled by an Arduino via manual knobs for maximum ease of use (Fig. 1b). The Raspberry Pi both controls the camera, which records the experiment, and communicates via serial port with the Arduino. Videos or images are captured via a Raspberry Pi Camera and can be processed through several programs. Here, we present the development of BioImageProcessing (BIO), an open-source software that can perform tracking of multiple unmarked individuals and be run both in on-line and off-line modes.

### 2.2 Illumination control

The arena is a laser cut Polymethyl methacrylate (PMMA) rectangular platform (120 × 120 mm) that can be located at different heights by using 3D printed spacers. This makes it possible to use different materials to change the diffusion of backlit infrared (IR) or white illumination. We provide a few designs that create different illumination or stimulation conditions by positioning IR and RGB LEDs at different levels (Fig. 1c). The dimensions of the arena provide enough surface to image small animals in a wide range of platforms such as petri dishes 60–100 mm or custom arenas such as the ones proposed by Koemans [4] or Gou [5].

The illumination modules have been designed to enable control of the orientation and translation (horizontal and vertical), as well as light intensity of all the light sources: Infrared for illumination of experiments in the dark, for example when performing optogenetics, and RGB for white light illumination and optogenetic stimulation. The device has a I^2^C port to connect an illuminance sensor that enables the user to characterize and adapt it to new light stimulation tools such as other RGB matrices, or LED displays. In addition, a spectral sensor can be connected but we did not use it in this manuscript because we found the resolution not useful for the experiments we were running.

**Fig. 2:**
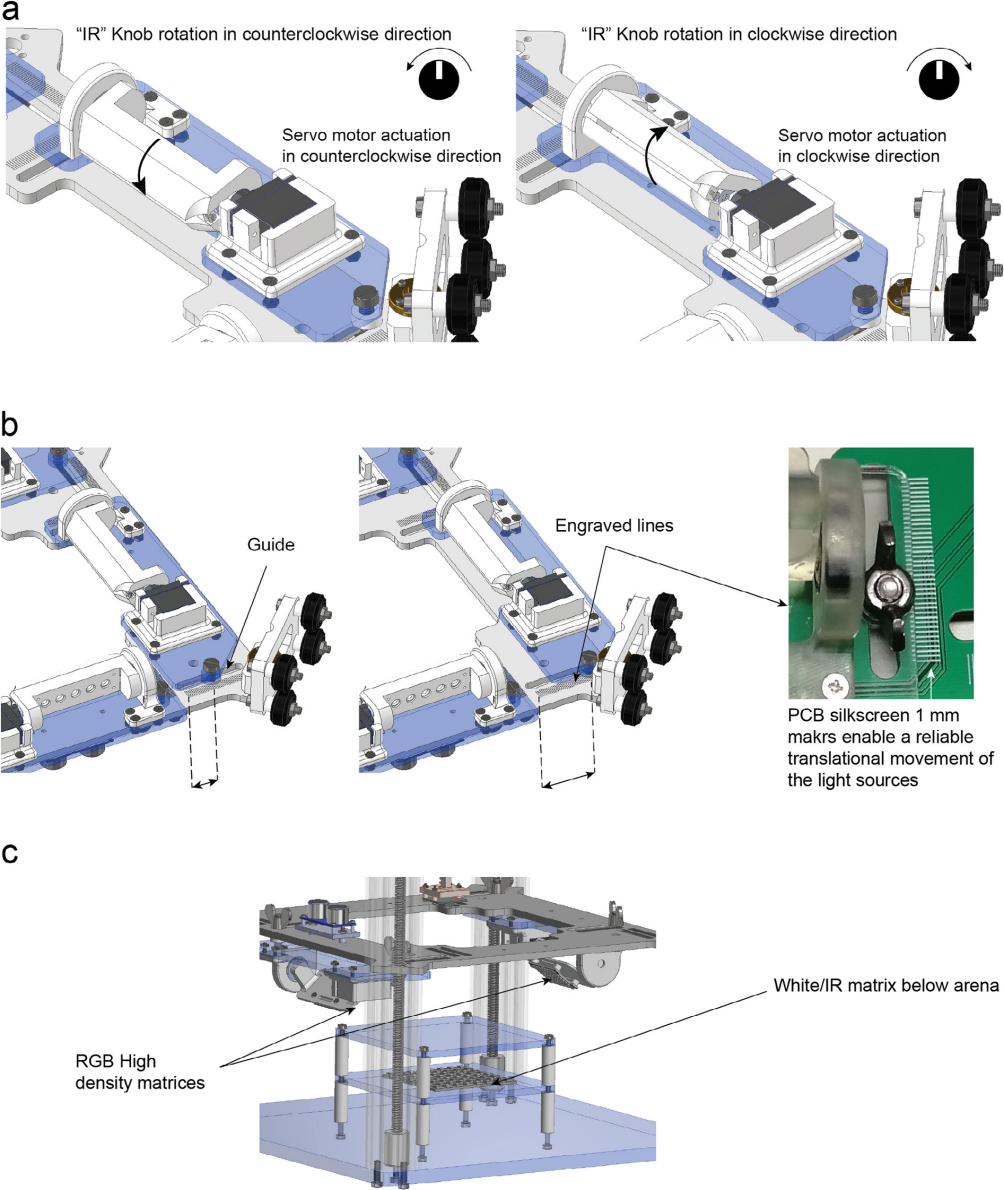
Movements that allow the customization of the device. a. Rotational movement of the infrared light sources are adjusted by rotating the potentiometer. b. The distance from all the light sources (RGB and IR) can be manually adjusted by untightening the M6 screws and sliding the light source platform forwards and backwards. c. Position of the RGB orientable matrices on the top platform and the arena underneath illumination.

The IR illumination is composed of four linear arrays surrounding the arena forming a square (hereafter ‘reflected IR modules’) and one matrix beneath the arena (hereafter ‘transmitted IR module’). Each linear array of the reflected IR modules is composed of five Infrared LED (AN5307B Stanley Electric, 940 nm IR LED, 5 mm) and five current limiting resistors (68 Ω, 1% tolerance), that provide the nominal forward current of I_F_≈50 mA, soldered on a custom PCB provided in the publication files. Each array is mechanically connected to a micro servo motor, SG90, that enables angle control within a range of approximately 2π/3 rad (120°) with 1° of resolution by rotating a 10 kΩ potentiometer located on the right side of the control board (Fig. 2a) (Supplementary video 1). The light intensity of the IR light sources can be controlled by an external 100 Ω, 5 W potentiometer. Each light source is mounted on a guide that provides manual translation adjustment (20 mm) by tweaking the tightness of two M6 wing nuts. A scale in millimetres is engraved next to both guides (Fig. 2b). The four light sources are held by a bigger platform that can move upwards and downwards (vertical translation movement) and therefore the height of the incident light can be changed.

An IR matrix is located beneath the arena to achieve backlight illumination when needed. It also has its own 100 Ω, 5 W potentiometer to adjust light intensity by limiting the current. Additionally, this matrix can be easily interchanged with a white LED array in order to achieve white light transmitted illumination (Fig. 2c).

**Fig. 3:**
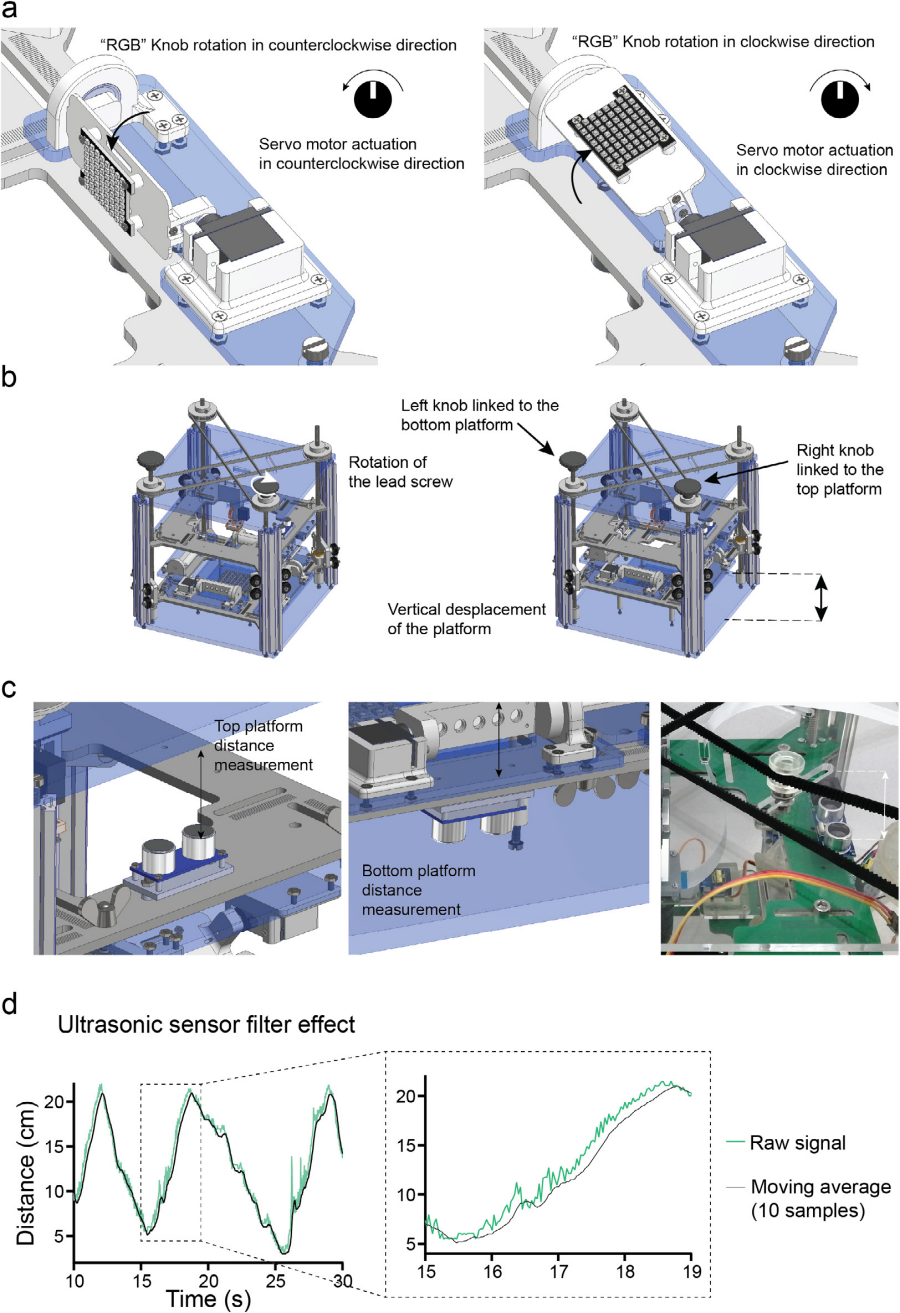
Customisation of light illumination. a. Rotational movement of the RGB matrices actuated by the analogue signal from the PCB potentiometer. b. The vertical position of the light source platforms (both IR and RGB) can be changed independently by rotating the two separate lead screws/ pulley systems. c. The ultrasonic sensors face the roof and the base of the OptoPi structure to measure the distance from each platform to its closest surface. These sensors determine the distance between the illumination platform and the OptoPi bottom and the distance between the stimulation platform and the OptoPi top. d. The moving average filter applied to the ultrasonic sensor signal results in less signal fluctuation making it easier for the user to read.

Overhead white light illumination can be achieved by employing the “optogenetic” modules with all RGB LEDs on at the same level for each colour. This illumination is achieved by controlling two RGB Adafruit Dotstar high density 8x8 grids (Adafruit 3444). These grids are mounted on a second platform identical to the one used for the infrared illumination sources. Therefore, they can be adjusted to the same degree of freedom as the IR linear arrays angle, and are controlled by a separate RGB knob. Horizontal and vertical positions can also be changed with similar knobs, as well as their light intensity with the corresponding potentiometer (Fig. 3a) (Supplementary video 2). The two platforms can be adjusted vertically by rotating a double GT2 timing belt-lead screw driven system (Fig. 3b) (Supplementary video 3). On each platform, an ultrasonic sensor HC - SR04 (Sparkfun 15569) measures that platform’s distance from the main structure: the illumination platform’s distance is relative to the bottom of the OptoPi and the stimulation platform relative to the top (Fig. 3c). The signal received from the ultrasonic sensors is filtered with a moving average filter. This improves the signal to noise ratio and eases the interpretation of the readings by the user (Fig. 3d).

### 2.3 Optogenetic modules

**Fig. 4:**
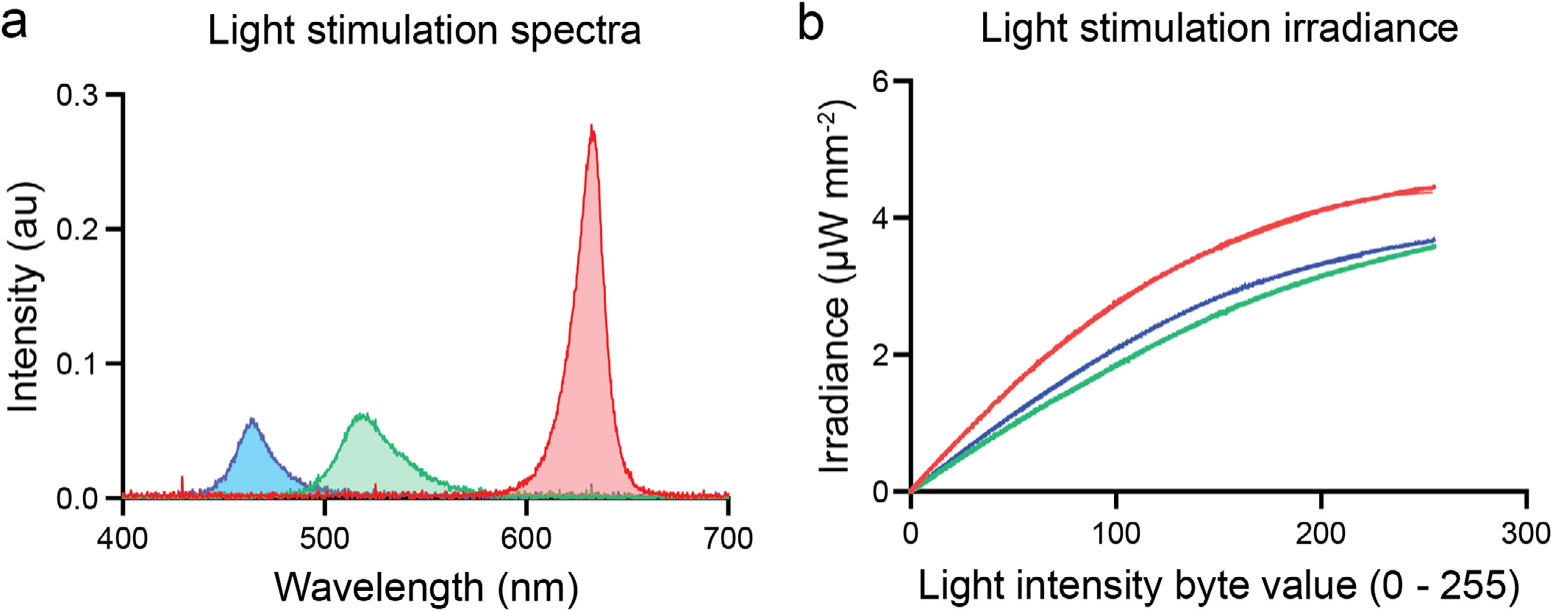
Optogenetic module characterization. a. Visible light spectrum of the RGB matrices measured with the Thorlabs CCS200/M compact spectrometer with the CCSB1 cosine corrector b. Irradiance measured on the setup using a platform with a calibrated Thorlabs S120VC photodiode connected to a PM100D compact power meter console.

The RGB illumination arrays can also be used as optogenetic modules. Optogenetics uses light to control the activity of cells that have been genetically modified to express light-sensitive ion channels. The optimal activation wavelengths of the most popularly used light-sensitive ion channels are 470, 490, 525 and 590 nm for GtACR2, ChR2^XXL^, GtACR1 and CsChrimson respectively [6], [7], [8]. In the present device we have measured the RGB matrices spectrum: 465,521 and 633 nm peak wavelengths using a Thorlabs CCS200/M compact spectrometer with the CCSB1 cosine corrector. These values fulfil the spectrum requirements for most of optogenetic experiments (Fig. 4a).

Most recent optogenetic actuators require a light irradiance of 1 µW/mm^2^ or less [6], [7], [8], [9], [10] to be activated. Our device can generate maximum irradiances of 4.45, 3.58, 3.69 µW/mm^2^ for red, green and blue respectively. It does so by using the two RGB high density grids (Fig. 4b). The measured average minimum change in irradiance is 0.015, 0.013, 0.012 µW/mm^2^ for the red, green, and blue LED channels respectively. These values have been measured using a calibrated Thorlabs S120VC photodiode and a PM100D power and energy meter. The measured residual irradiance (Brightness byte value 0) of the device is 1.25·10^-4^ µW/mm^2^.

Finally, the device has the capability to perform light intensity (TSL2561 Digital Luminosity Lux Light Sensor Module) measurements in the same platform arena where experimental animals will be placed. This enables precise measurement of the light stimulation intensity for any specific position or configuration of the RGB matrices. This feature, together with positional readings from the ultrasound sensors and motor angles of the LEDs, enables the precise recording of stimulation conditions. This includes the irradiance of the behavioural arena, allowing the quick reproduction of experimental settings within and across labs.

The main control shield is a custom PCB which has all the connections and required elements to drive the device with the pins used on the Arduino sketches.

### 2.4 Recording settings

**Fig. 5:**
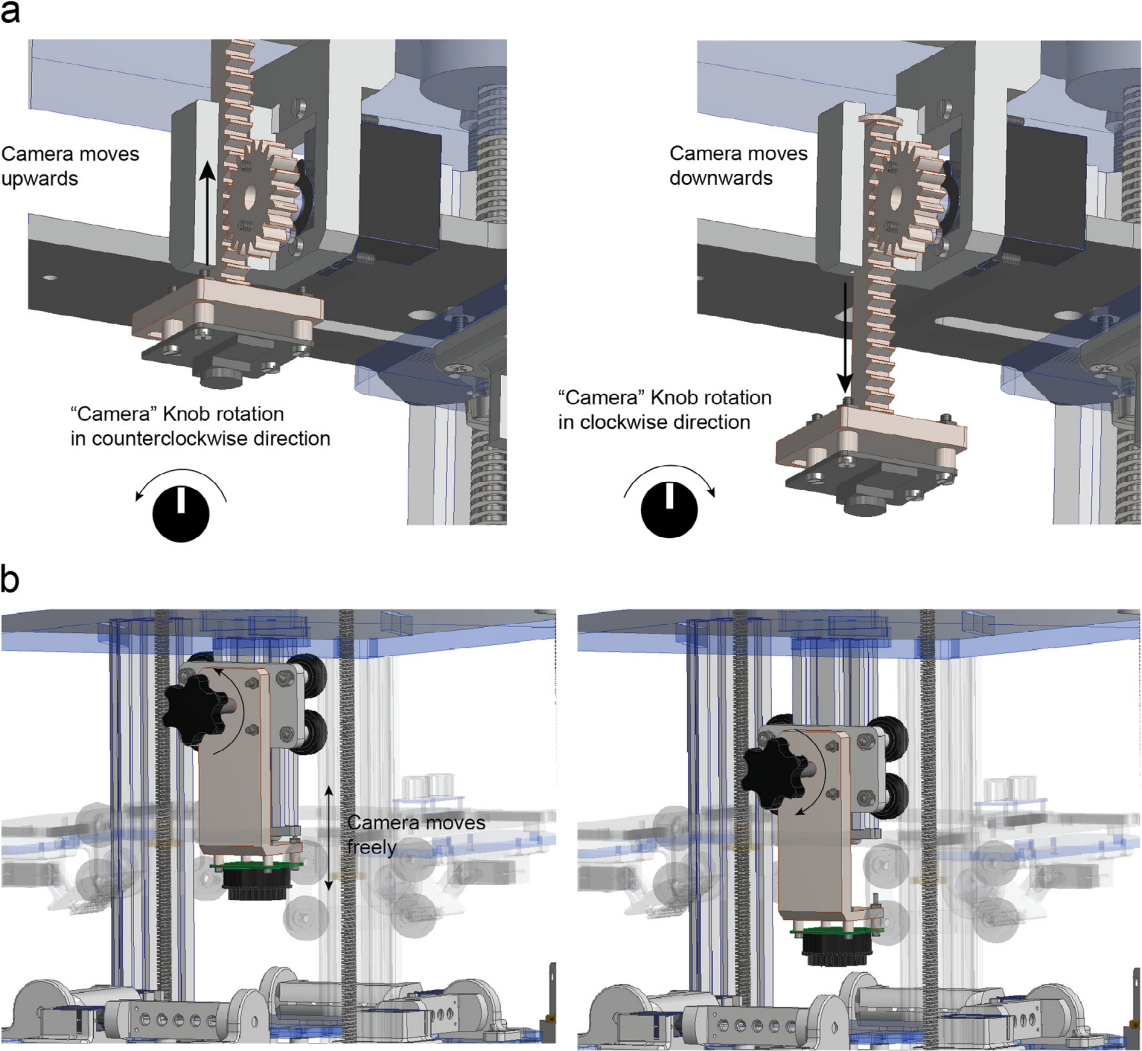
Camera vertical movement. a. The Camera knob actuates the servo motor of the camera translational stage moving the camera up or down and helping the user to select the right imaging distance and the right field of view. b. Manual camera stage adjustment process by tightening or untightening the thumb screw.

The OptoPi has been tested using both the V2 and HQ Raspberry Pi camera units. Video recordings made for subsequent tracking were acquired with the Raspberry Pi HQ camera equipped with a Raspberry Pi HQ camera 6 mm 3MP lens. For optogenetic experiments with IR illumination this lens can be fitted with a mounted filter that only allows the passage of infrared (IR) spectrum of the incident light (MIDOPT FIL LP830/27). The Raspberry Pi HQ camera’s IR filter can be removed according to the instructions provided by the manufacturer to make it IR-sensitive. The camera can be mounted either in a electromechanical stage or a purely mechanical stage. The electromechanical stage consists of a SG90 micro servo motor (Tower pro) mounted on a rack-spur gear linear stage that converts the rotational movement of the servo motor into a linear movement (Fig. 5a). The distance from the camera to the arena can be adjusted within a range of approximately 50 mm (Supplementary video 4). The purely mechanical stage can be built based on a cart running on an aluminium rail by untightening a thumb screw (Fig. 5b). We found that while the electromechanical stage provided easier experimental modification of the camera z position, it incurred in small xy translation (jiggling) which could be impractical for some experiments. In turn, the mechanical stage is extremely precise, both in z and xy, but positioning it requires delicate manual adjustment.

The Raspberry Pi requires a micro Secure Digital (SD) card for its OS and data recording. The Raspberry Pi 4 support micro-SD cards from 8 GB up to 64 GB, using the FAT32 file system. For high quality video recording we recommend using class U3 cards.

### 2.5 Tracking options

Videos in this manuscript were acquired either using custom written python scripts on a Raspberry Pi running Raspbian OS or directly via BioImageOperation on the same Raspberry Pi running Ubuntu (see Operation instructions). Videos were recorded in h264 format. Here we present a tracking software called BioImageOperation (BIO, https://github.com/folterj/BioImageOperation). BIO is a light-weight solution allowing real-time tracking of many individuals of similar or different types, with cross-platform support including low-cost hardware such as Raspberry Pi. BIO tracks multiple animals by using a combination of location and trajectory information, in addition uses time-independent characteristics to retain identity. These are temporal mean values of area size and length across the primary axis.

The main advantage of BIO is its light-weight, which means that can be combined with inexpensive hardware for live tracking, and its capacity to keep relatively accurate (see table below) identity of multiple animals simultaneously even with Low quality (LQ) video image (i.e. when each animal is represented by few pixels). For illustration of its performance, we directly compared BIO with two recently published and widely used trackers, and their features and statistics are summarised in the tables below. Briefly, BIO performs comparably better than other trackers when dealing with low quality videos/live feeds. This is an important feature because the result of low-cost cameras when imaging a large field of view to track many animals is often a low-quality video. In turn, other tracking softwares can keep individual’s identity more accurately than BIO when used on high quality (HQ) videos. However, the computational requirements of these softwares prevent them from being used in on-line mode on a Raspberry Pi.

## 3 Features & support

**Table:** 

Software	Cross-platform	Low-cost hardware	Live tracking	Unmarked subjects	HQ identity preservation	LQ identity preservation
idtracker.ai[11]	✓			✓	98.95%*	–
TRex [12]	✓		✓	✓	99.07%*	68.84%
BIO	✓	✓	✓	✓	93.58%	73.83%

* Reported values.

- Tracking failed.

HQ: high quality videos.

LQ: low quality videos.

Source & tracking statistics.

**Table:** 

Label	Species	Resolution	FPS	# individuals	# pixels per individual	RAM usage	Offline processing: Laptop* [FPS]	Online processing Pi** [FPS]
HQ	Zebrafish (Danio rerio)	3584x3500 (12.5MP)	32	100	∼500	∼250 MB	10.5	–
LQ	Fruit fly larva (Drosophila melanogaster)	1000x1200 (1.2MP)	30	8	∼60	∼100 MB	124.0	40.4^#^

* Laptop: Dell Latitude 5420 (not using GPU).

** Pi: Raspberry Pi 4B (RPi 2018) 4 GB.

^#^ Value when not limited by fixed camera frame speed of 30 FPS.

- Unknown.

Design files.

Design Files Summary.

**Table:** 

Design file name	File type	Open source license	Location of the file
*Design files (Step and Autodesk inventor).zip*	*Autodesk inventor assembly and parts and STEP assembly*	*GPL3.0*	https://osf.io/pu8f5/files/osfstorage
*Arena*	*DXF*	*GPL3.0*	https://osf.io/pu8f5/files/osfstorage
*Calibration Arena*	*DXF*	*GPL3.0*	https://osf.io/pu8f5/files/osfstorage
*Base light source*	*DXF*	*GPL3.0*	https://osf.io/pu8f5/files/osfstorage
*Platform spacer*	*DXF*	*GPL3.0*	https://osf.io/pu8f5/files/osfstorage
*Base*	*DXF*	*GPL3.0*	https://osf.io/pu8f5/files/osfstorage
*Roof*	*DXF*	*GPL3.0*	https://osf.io/pu8f5/files/osfstorage
*Arena spacer 20 mm*	*STL*	*GPL3.0*	https://osf.io/pu8f5/files/osfstorage
*Arena spacer 30 mm*	*STL*	*GPL3.0*	https://osf.io/pu8f5/files/osfstorage
*Linear actuator servo attachment*	*STL*	*GPL3.0*	https://osf.io/pu8f5/files/osfstorage
*Raspberry Pi HQ Camera rack*	*STL*	*GPL3.0*	https://osf.io/pu8f5/files/osfstorage
*Raspberry pi camera V2 rack*	*STL*	*GPL3.0*	https://osf.io/pu8f5/files/osfstorage
*Spur gear*	*STL*	*GPL3.0*	https://osf.io/pu8f5/files/osfstorage
*LED linear array holder*	*STL*	*GPL3.0*	https://osf.io/pu8f5/files/osfstorage
*Matrix holder*	*STL*	*GPL3.0*	https://osf.io/pu8f5/files/osfstorage
*Micro servo holder*	*STL*	*GPL3.0*	https://osf.io/pu8f5/files/osfstorage
*Shaft attachment*	*STL*	*GPL3.0*	https://osf.io/pu8f5/files/osfstorage
*Cart spacer*	*STL*	*GPL3.0*	https://osf.io/pu8f5/files/osfstorage
*Cart*	*STL*	*GPL3.0*	https://osf.io/pu8f5/files/osfstorage
*M6 spacer*	*STL*	*GPL3.0*	https://osf.io/pu8f5/files/osfstorage
*Ultrasonic sensor holder*	*STL*	*GPL3.0*	https://osf.io/pu8f5/files/osfstorage
*Belt tensioner*	*STL*	*GPL3.0*	https://osf.io/pu8f5/files/osfstorage
*Lead screw manual adjuster*	*STL*	*GPL3.0*	https://osf.io/pu8f5/files/osfstorage
*Pulley*	*STL*	*GPL3.0*	https://osf.io/pu8f5/files/osfstorage
*Raspberry Pi HQ holder V2 (Optional for mechanical camera stage)*	*STL*	*GPL3.0*	https://osf.io/pu8f5/files/osfstorage
*Camera cart V2 (Optional for mechanical camera stage)*	*STL*	*GPL3.0*	https://osf.io/pu8f5/files/osfstorage
*Spacer (Optional for mechanical camera stage)*	*STL*	*GPL3.0*	https://osf.io/pu8f5/files/osfstorage
*Stop (Optional for mechanical camera stage)*	*STL*	*GPL3.0*	https://osf.io/pu8f5/files/osfstorage
*Control Board Shield PCB*	*Gerber files*	*GPL3.0*	https://osf.io/pu8f5/files/osfstorage
*IR LED Array PCB*	*Eagle, Gerber*	*GPL3.0*	https://osf.io/pu8f5/files/osfstorage
*IR Underneath Matrix PCB*	*Eagle, Gerber*	*GPL3.0*	https://osf.io/pu8f5/files/osfstorage
*Platform PCB*	*Gerber files*	*GPL3.0*	https://osf.io/pu8f5/files/osfstorage
*Camera servo Adaptor PCB*	*Gerber files*	*GPL3.0*	https://osf.io/pu8f5/files/osfstorage
*I2C Adaptor PCB*	*Gerber files*	*GPL3.0*	https://osf.io/pu8f5/files/osfstorage
Servo test	Arduino IDE	*GPL3.0*	https://osf.io/pu8f5/files/osfstorage
OptoPi	Arduino IDE	*GPL3.0*	https://osf.io/pu8f5/files/osfstorage
OptoPi_ISR	Arduino IDE	*GPL3.0*	https://osf.io/pu8f5/files/osfstorage
video_annotations_sync	Python	*GPL3.0*	https://osf.io/pu8f5/files/osfstorage
record_video_interrupts_ISR	Python	*GPL3.0*	https://osf.io/pu8f5/files/osfstorage

## 4 Bill of materials

Bill of Materials.

**Table:** 

Designator	Component	Number	Cost per unit -currency	Total cost –currency	Source of materials	Material type
*Structure acrylic base*	*Base.dxf*	*1*	*£14.31*	*£14.31*	*Sheet plastics*	*PMMA (10 mm thickness)*
*Structure acrylic roof*	*Roof.dxf*	*1*	*£14.31*	*£14.31*	*Sheet plastics*	*PMMA (10 mm thickness)*
*Arena*	*Arena.dxf*	*2*	*£1.12*	*£2.24*	*Sheet plastics*	*PMMA (5 mm thickness)*
*Arena with illuminance sensor attachment*	*Calibration Arena.dxf*	*1*	*£1.12*	*£1.12*	*Sheet plastics*	*PMMA (5 mm thickness)*
*Illumination and light source platform*	*Platform spacer.dxf*	*2*	*£14.31*	*£28.62*	*Sheet plastics*	*PMMA (10 mm thickness)*
*Light source base*	*Base light source.dxf*	*6*	*£0.40*	*£2.40*	*Sheet plastics*	*PMMA (5 mm thickness)*
*GT2 pulley*	*Pulley*	*4*	*£3.92*	*£15.68*	*–*	*Formlabs Clear Resin*
*Lead screw knob*	*Lead screw manual adjuster*	*2*	*£2.54*	*£5.08*	*–*	*Formlabs Clear Resin*
*Belt tensioner*	*Belt tensioner*	*2*	*£0.12*	*£0.24*	*–*	*Formlabs Clear Resin*
*Arena spacer 30 mm long*	*Arena spacer 30 mm*	8	*£0.17*	*£1.36*	–	*Formlabs Clear Resin*
*Arena spacer 20 mm long*	*Arena spacer 20 mm*	8	*£0.11*	*£0.88*	–	*Formlabs Clear Resin*
*Light sources platform cart*	*Cart*	4	*£2.49*	*£9.96*	–	*Formlabs Clear Resin*
*Cart spacer*	*Cart spacer*	16	*£0.04*	*£0.64*	–	*Formlabs Clear Resin*
*M6 screw spacer*	*M6 spacer*	12	*£0.05*	*£0.60*	–	*Formlabs Clear Resin*
*Distance ultrasonic sensor holder*	*Ultrasonic sensor holder*	2	*£0.42*	*£0.84*	–	*Formlabs Clear Resin*
*Micro servo holder*	*Micro servo holder*	6	*£1.94*	*£11.64*	–	*Formlabs Clear Resin*
*Light source/optogenetic holder rotary joint*	*Shaft attachment*	6	*£0.61*	*£3.66*	–	*Formlabs Clear Resin*
*Optogenetic matrix holder*	*Matrix holder*	2	*£0.68*	*£1.36*	–	*Formlabs Clear Resin*
*Infrared light source PCB holder*	*LED linear array holder*	4	*£2.13*	*£12.78*	–	*Formlabs Grey Resin*
*Camera gear*	*Spur gear*	1	*£0.15*	*£0.15*	–	*Formlabs Clear Resin*
*Raspberry Pi HQ holder*	*Raspberry Pi HQ Camera rack*	1	*£1.05*	*£1.05*	–	*Formlabs Clear Resin*
*Camera control stage body*	*Linear actuator servo attachment*	1	*£3.43*	*£3.43*	–	*Formlabs Clear Resin*
*Raspberry pi camera V2 holder*	*Raspberry pi camera V2 rack*	1	*£0.55*	*£0.55*	–	*Formlabs Clear Resin*
*Raspberry Pi HQ holder V2 (Optional for mechanical camera stage)*	*Raspberry Pi HQ holder V2*	1	*£0.6*	*£0.6*	–	*Formlabs Clear Resin*
*Camera cart V2 (Optional for mechanical camera stage)*	*Camera cart V2*	1	*£0.50*	*£0.50*		*Formlabs Clear Resin*
*Spacer (Optional for mechanical camera stage)*	*Spacers*	4	*£0.05*	*£0.20*		*Formlabs Clear Resin*
*Stop (Optional for mechanical camera stage)*	*Stop*	1	*£0.10*	*£0.10*		*Formlabs Clear Resin*
*Camera longpass filter*	*LP830 Near-IR Longpass Filter*	1			Midopt	
*Main control board shield PCB*	*Control Board PCB*	1	∼£3	£3	JLCPCB	
Infrared LED linear array PCB	*IR LED Array PCB*	4	∼£1.5	£6	JLCPCB	
Underneath matrix PCB	*IR Underneath Matrix PCB*	1	∼£3	£3	JLCPCB	
Light sources Platform PCB	*Platform spacer PCB*	4	∼£5	£20	JLCPCB	
Camera servo motor adaptor	Camera servo motor adaptor PCB	1	∼£1.5	£1.5	JLCPCB	
I2C adaptor	I2C adaptor PCB	1	∼£1.5	£1.5	JLCPCB	
Barrel jack PCB connectors	Barrel jack PCB connectors	6	∼£0.2	£1.2	Amazon	
Screw terminals to barrel jack connectors	Screw terminals to barrel jack connectors	1	£0.6	£0.6	Amazon	
Ethernet PCB connectors	Ethernet PCB connectors	8	£0.33	£2.64	Farnell	
Barrel jack cables	Barrel jack cables	2	£7.95	£7.95	Amazon	
Ethernet cables	Ethernet cables	4	£4.04	£16.16	Amazon	
Control board Pushbutton	Pushbutton	2	£3.49	£3.49	Amazon	
Arduino Mega 2560 development board	*Arduino Mega 2560*	1	£35.70	£35.70	RS Components	
Raspberry Pi micro computer	*Raspberry pi 4B (RPi 2018) 4 GB*	1	£65.51	£65.51	RS Components	
Raspberry Pi SD memory	*8*–*64 GB micro SD*	1	£11.49	£11.49	Amazon	
Raspberry Pi HQ camera	*Raspberry pi camera HQ*	1	£47.27	£47.27	RS Components	
Raspberry pi camera lens	*Raspberry pi camera lens 6 mm wide angle*	1	£ 24.90	£ 24.90	The PiHut	
Light angle control rotary servo motors	*SG90s servo motors*	7	£4.24	£29.68	Rapid Electronics	
Distance measuring ultrasonic sensors	*Ultrasonic sensor HC-SR04*	2	£3.99	£7.98	Amazon	
Optogenetic stimulation matrix	*Dotstar RGB 8x8 Grid*	2	£18.06	36.12	Adafruit	
GT2 timing belt	*GT2 belt 6x852 mm*	2	£4.79	£9.59	Amazon	
Infrared LED	*IR LED 940 nm*	36	£0.302	£10.872	RS Components	
Resistor for the IR LED	*Resistor 68 Ω, 0.25 W ± 1%*	36	£0.275	£9.9	RS Components	
Big potentiometer	*Potentiometer 100 Ω, 5 W*	2	£3.10	£6.20	RS Components	
Small potentiometer	*Potentiometer 100 kΩ, 1 W*	4	£1.11	£4.44	Mouser	
M4 threaded insert	*M4 threaded insert*	18	£0.114	£2.052	RS Components	
Ball bearing	*Bearing 19 mm O.D*	6	£2.53	£15.18	RS Components	
Rocker switch	*ON/OFF switch*	1	£1.615	£1.615	RS Components	
Power supply	*Power supply 5 V, 30 W*	1	£29.02	£29.02	RS Components	
Lead screw coupler	*5 mm to 8 mm coupler*	4	£4.99	£19.98	Amazon	
Lead screw	*T8 Lead Screw with brass nut*	4	£15.29	£61.16	Amazon	
Aluminium extrusion	*2040 v-slot aluminium extrusion*	4	£8.88	£35.52	Amazon	
Cart wheel	*Wheel bearing*	16	£1.1	£17.60	Amazon	
Illuminance I2C sensor	*TSL2561 Digital Luminosity Lux Light Sensor Module*	1	£6.24	£6.24	Amazon	
Camera rail (Optional for the mechanical camera stage)	2040 Aluminium Extrusion 100 mm	1	£11.99	£11.99	Amazon	
Thumb screw(Optional for the mechanical camera stage)	M8 Thumb screw	1	£0.65	£0.65	Amazon	
Wheel bearing(Optional for the mechanical camera stage)	Wheel bearing	4	£1.4	£5.6	Amazon	
Spectrometer I2C sensor (Optional)	*Adafruit AS7341 Spectrometer*	1	£ 14.50	£ 14.50	The PiHut	

## 5 Build instructions

This section has the following subsections:

5.1. Generate all mechanical pieces.

5.2. Assembly of the illumination infrared LED arrays.

5.3. Assembly of the RGB Matrix holder.

5.4. Camera linear stage assembly.

5.5. Arena assembly.

5.6. Cart assembly.

5.7. Platform assembly.

5.8. Device final mechanical assembly.

5.9. Soldering.

5.10. Electrical connections.

5.11. Ease of manufacture.

### 5.1 Generate all mechanical pieces.

**Fig. 6:**
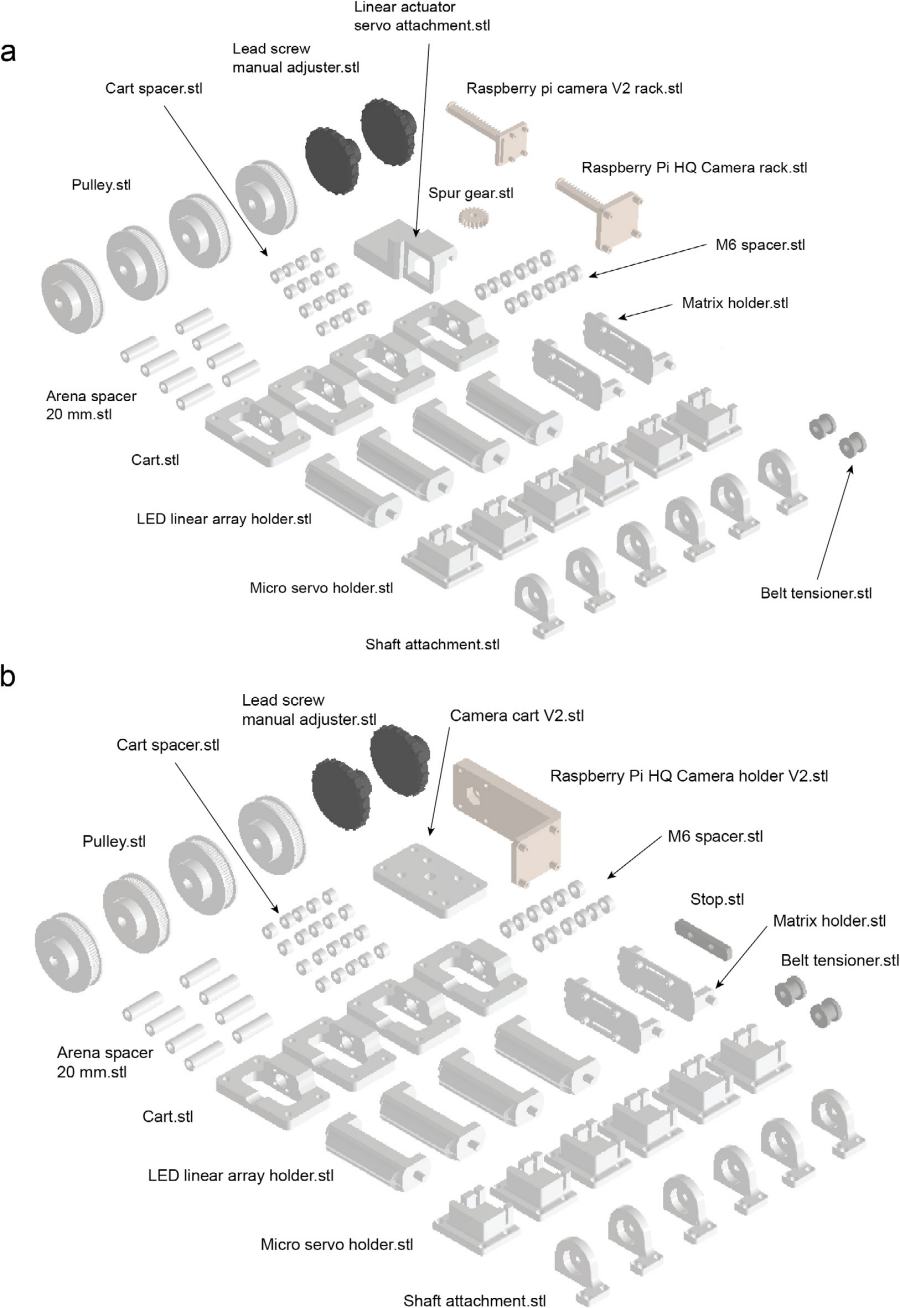
OptoPi components to 3D print. a. With the electromechanical camera stage. b. With the manual camera stage.

1- Print all required 3D printed pieces (Fig. 6). In our case we used a Formlabs Form 3 3D printer with 100 µm resolution and Clear and Gray resins.

**Fig. 7:**
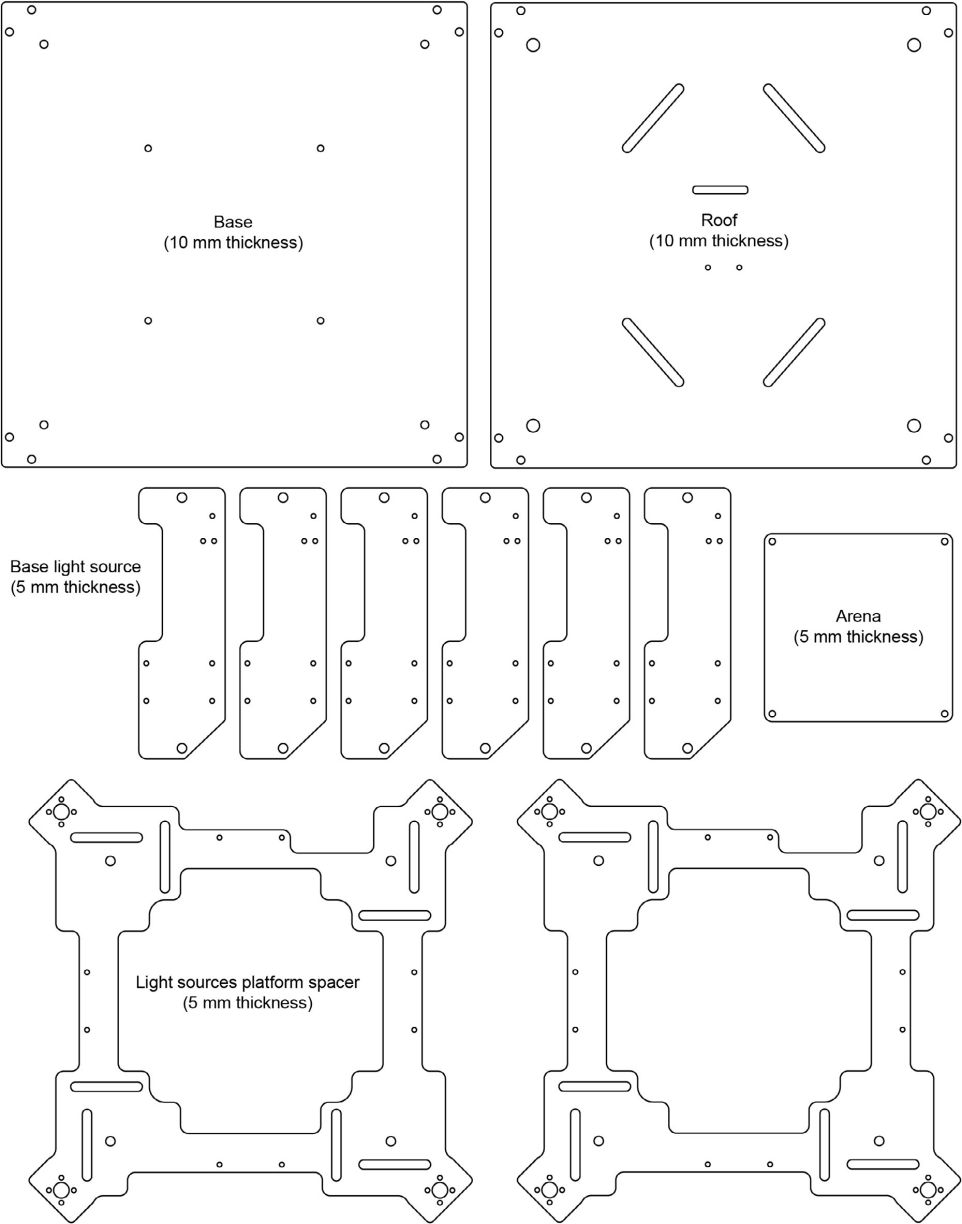
OptoPi components to laser cut.

2- Laser cut all of the required pieces (Fig. 7). In our case we used a Universal laser systems VLS3.50 laser cutter and PMMA sheets (5 and 10 mm thickness).

### 5.2 Assembly of the illumination infrared LED arrays

**Fig. 8:**
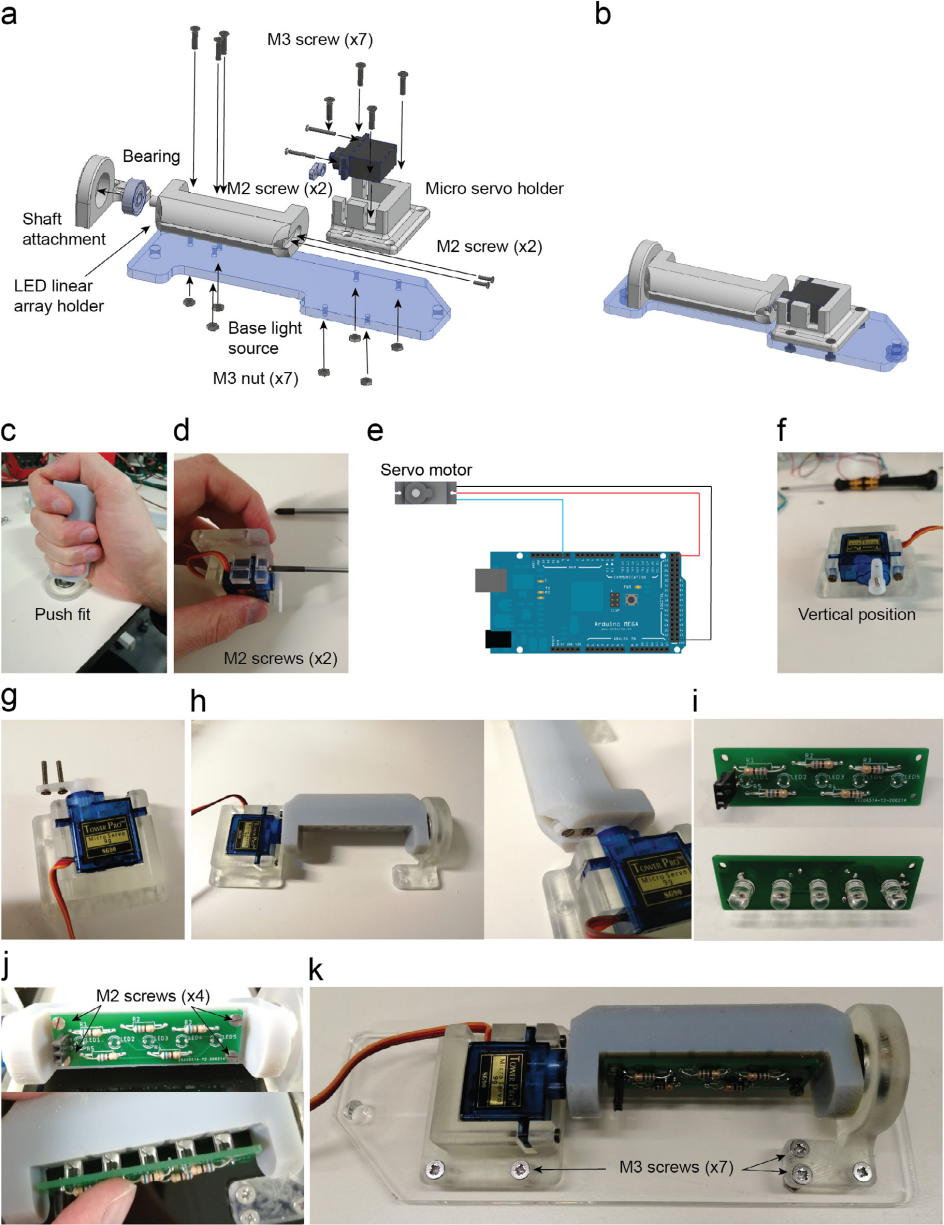
Infrared LED array assembly. a. Exploded view of the illumination infrared LED arrays. b. Assembled view of the illumination infrared LED arrays (Supplementary video 5). c. LED linear array holder inserted into the bearing. d. Servo motor attached to holder with two M2 screws. e. SG90s servo motor connection for its calibration. f. Position of the servo’s arm required for the calibration. g. Two M2 screws are placed on the servo lever after drilling two holes to increase their diameter. h. Assembly completed and detail of the two M2 screws threaded into the LED linear array holder part. i. Infrared LED soldered on the LED array PCB. 68 Ω resistors are soldered onto the other side of the PCB which is attached with four M2 screws threaded on the plastic. j. The PCB is attached with 4 M2 screws on the 3D printed part. The LEDs are inserted into five holes by pushing them through. k. The assembly is completed by attaching it on the base light source part with seven M3 screws and their respective nuts.

Insert the ball bearing on the Shaft attachment. The shaft of the LED linear array holder fits on the bearing just by push fit (Fig. 8c).

Attach the micro servo on the micro servo holder with two M2 screws (Fig. 8d).

Connect the servo motor to the Arduino 5 V, GND and Pin 9 (Fig. 8e).

Upload the program *Servo_test* to the Arduino board.

Connect the Arduino with the USB cable.

Unscrew the Servo arm.

Place the servo arm vertical (Fig. 8f) The vertical position can vary slightly from one servo motor to the other. This can be adjusted afterwards adding or subtracting one degree to the particular servo motor that is not aligned with the others.

Insert the two M2 screws on the servo arm (Fig. 8g).

Do the same with all the servo motors of the setup that are moving RGB LED matrices or IR LED arrays.

All the steps have to be done for the seven servo motors attached to their holders.

Then insert two M2 screws on the servo white arm (Fig. 8g).

Screw this two M2 screws with the LED holder (Fig. 8h).

Solder the IR LED 940 nm and the 68 Ω resistors on the LED Array PCB (Fig. 8i).

Insert the LED array on their holder and insert 4 screws to hold the PCB (Fig. 8j).

Attach the previous assembly with the light source base laser cut part (Fig. 8k).

Repeat the process for each of the four LED linear array.

### 5.3 Assembly of the RGB matrix holder

**Fig. 9:**
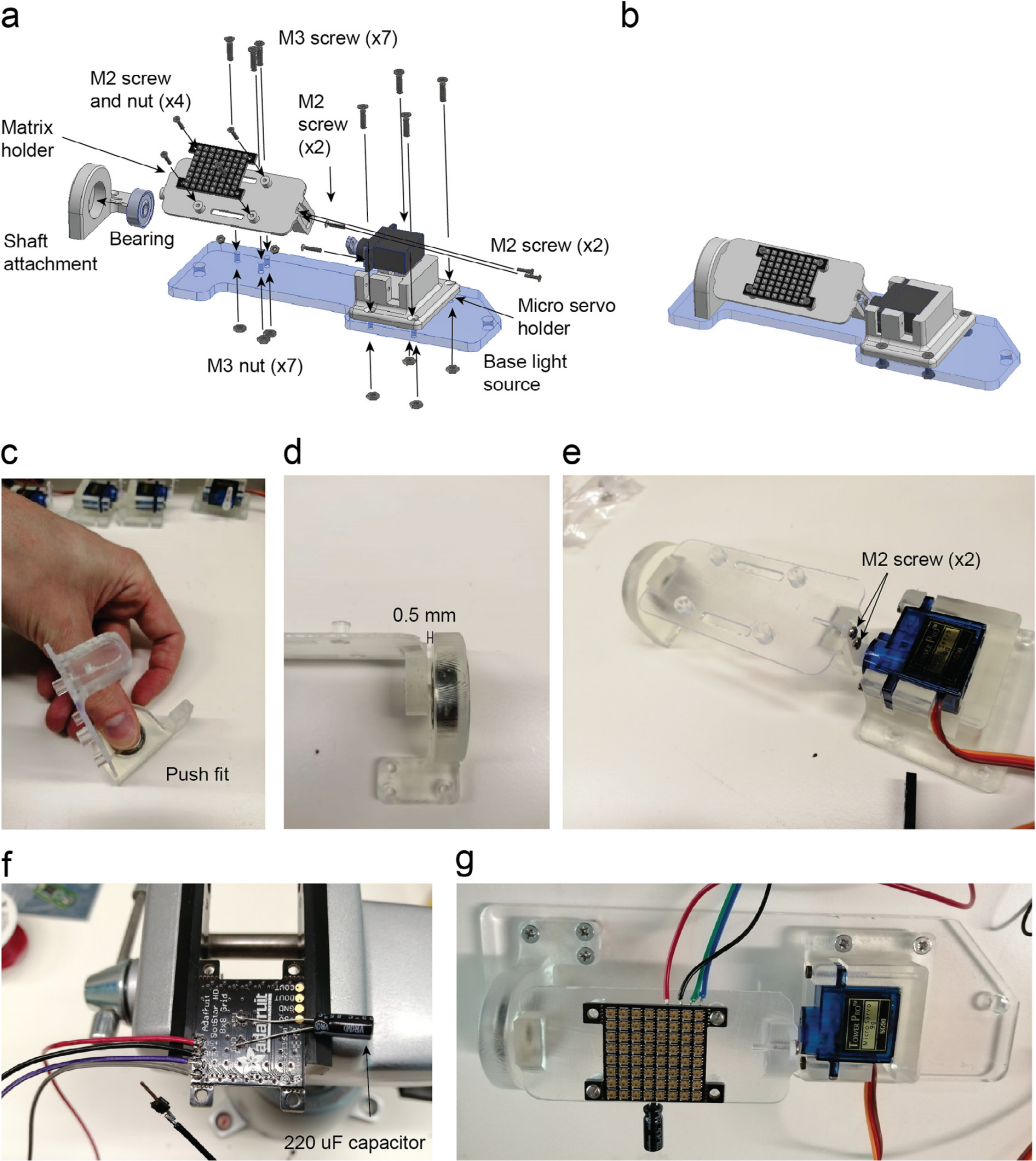
RGB matrix holder assembly. a. Exploded view of the RGB matrix holder. b. Assembled view of the RGB matrix holder (Supplementary video 6). c. The shaft of the matrix holder fits on the bearing by push fit. d. Assembled RGB matrix holder with the detail in the space between the bearing and the 3D printed part to avoid friction. e. Assembled RGB matrix with two M2 screws. f. Detail in the wires soldered on the RGB grid pads as well as the 220 µF capacitor that comes with the grid. g. The assembly is completed by attaching it onto the base light source part with seven M3 screws and their respective nuts.

Insert the ball bearing on the Shaft attachment (Fig. 9a-c).

Insert the shaft of the RGB LED Matrix holder on the bearing hole leaving a 0.5 mm distance to avoid friction (Fig. 9c-d).

Attach the micro servo on the micro servo holder with two M2 screws as done with the Infrared holders before.

Insert the two M2 screws on the servo arm.

Screw these two screws with the LED holder (Fig. 9e).

Solder the 220 µF capacitor and the Vcc, GND, Data and Clock wires on the matrix pads (Fig. 9f).

Attach the Matrices on the holder with four M2 screws (Fig. 9f).

Attach the system with the Base light source (Fig. 9g).

### 5.4 Camera linear stage assembly

#### 5.4.1 Electromechanical version

**Fig. 10:**
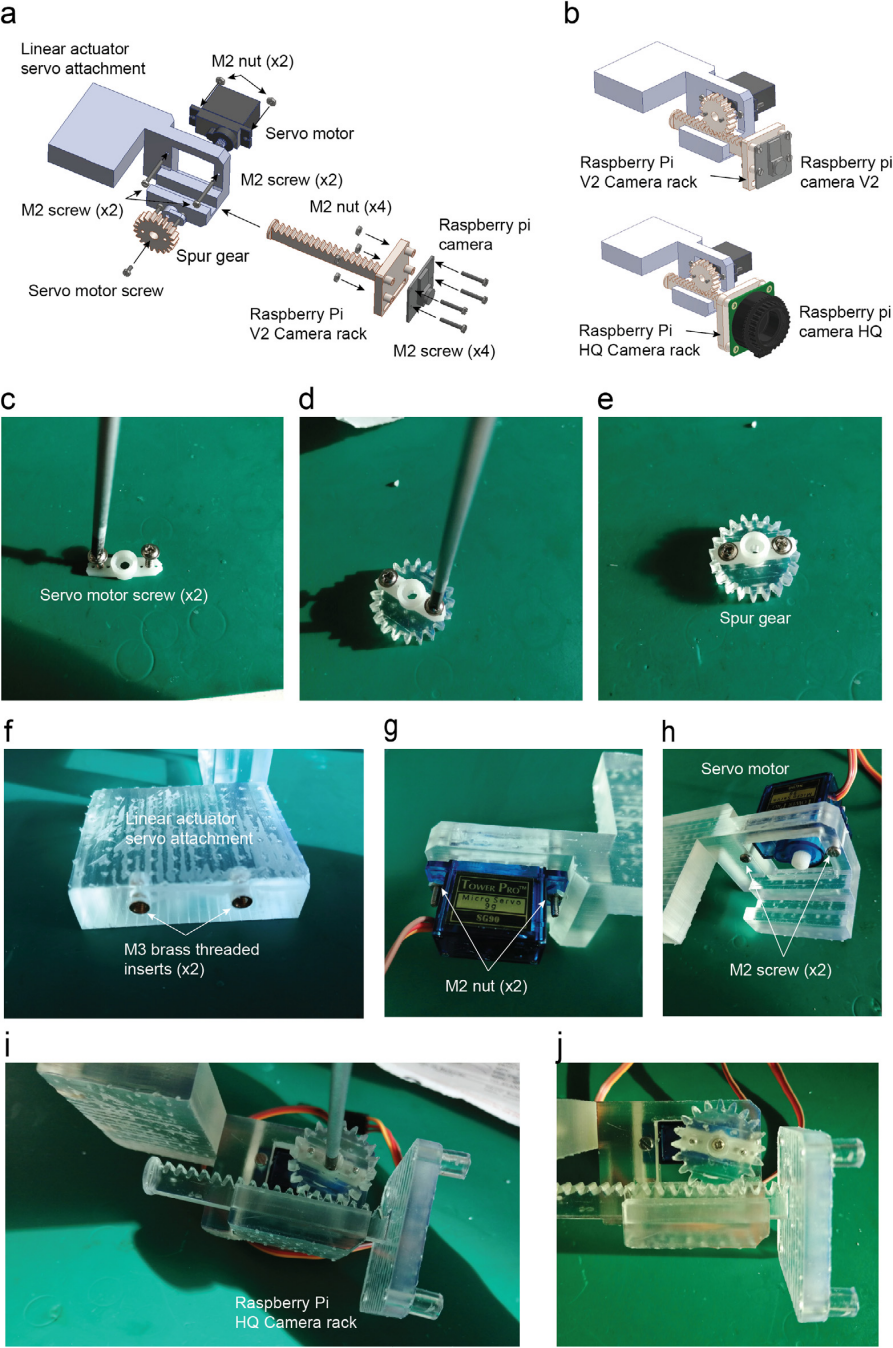
Electromechanical camera linear stage assembly. a. Exploded view. b. Assembled view of both Raspberry Pi camera versions (V2 and HQ) 3D printed holders (Supplementary video 7). c. The servo motor arm is shortened by cutting both sides at the level of the third hole. Then the servo motor screws are screwed in the second hole on each side of the servo motor. d. The arm is placed over the spur gear with the holes coincident. e. The servo arm and the spur gear are attached with the screws. f. Two M3 threaded inserts are inserted by applying pressure before curing the resin. g. The servo motor is attached using two M2 screws with M2 nuts. h. Detail of the M2 screws on the front view of the assembly. i. Spur gear assembly, the rack is placed in the channel and then the spur gear is attached making the teeth coincident and keeping the maximum travel range. j. The servo motor screw holds the spur gear as a final step.

Cut the servo motor white adaptor which has two symmetric sides leaving just the same diameter as the internal diameter of the spur gear (Fig. 10c-e).

Insert the two M3 threaded inserts with the linear actuator servo attachment just applying pressure manually (Fig. 10f).

Attach the servo motor with the linear actuator servo attachment using two M2 screws and nuts (Fig. 10g-h).

Place the rack on the guide and the spur gear with their teeth coincident and use the servo motor shaft screw to attach the spur gear with the servo motor. Make sure the servo motor has the whole rotational range in a useful linear range of motion of the rack (Fig. 10i-j).

#### 5.4.2 Mechanical version

Use the M5 tap tool to thread the holes on both sides of the aluminium extrusion.

**Fig. 11:**
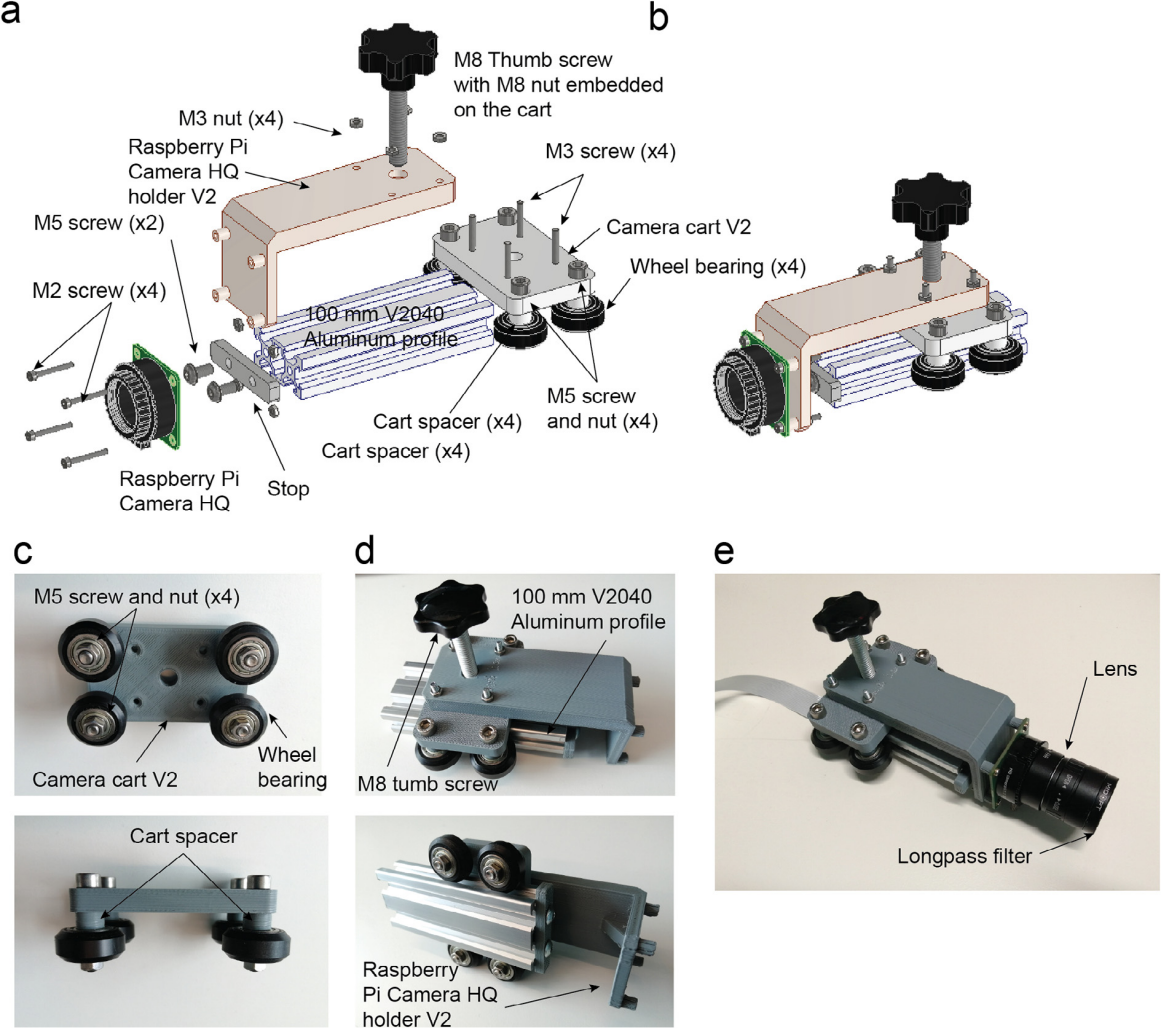
Mechanical camera linear stage assembly. a. Exploded view. b. Assembled view. c. Camera cart assembly. d. Linear rail assembly. e. Camera mounted on the stage.

Assemble the cart with the camera cart wheels using four M5 screws and nuts and four spacers in between (Fig. 11a-c).

Attach the Raspberry Pi camera HQ holder V2 with the camera cart using four (Fig. 11a-b).

Slide the cart in the aluminium extrusion rail and use two M5 screws to attach the stop in the end of the rail (Fig. 11d).

Attach the Raspberry Pi HQ camera with its lens and filter (Fig. 11e).

### 5.5 Arena assembly

Use M4 grub screws for each level of the arena you want and regular M4 screws for the top (Fig. 11a).

**Fig. 12:**
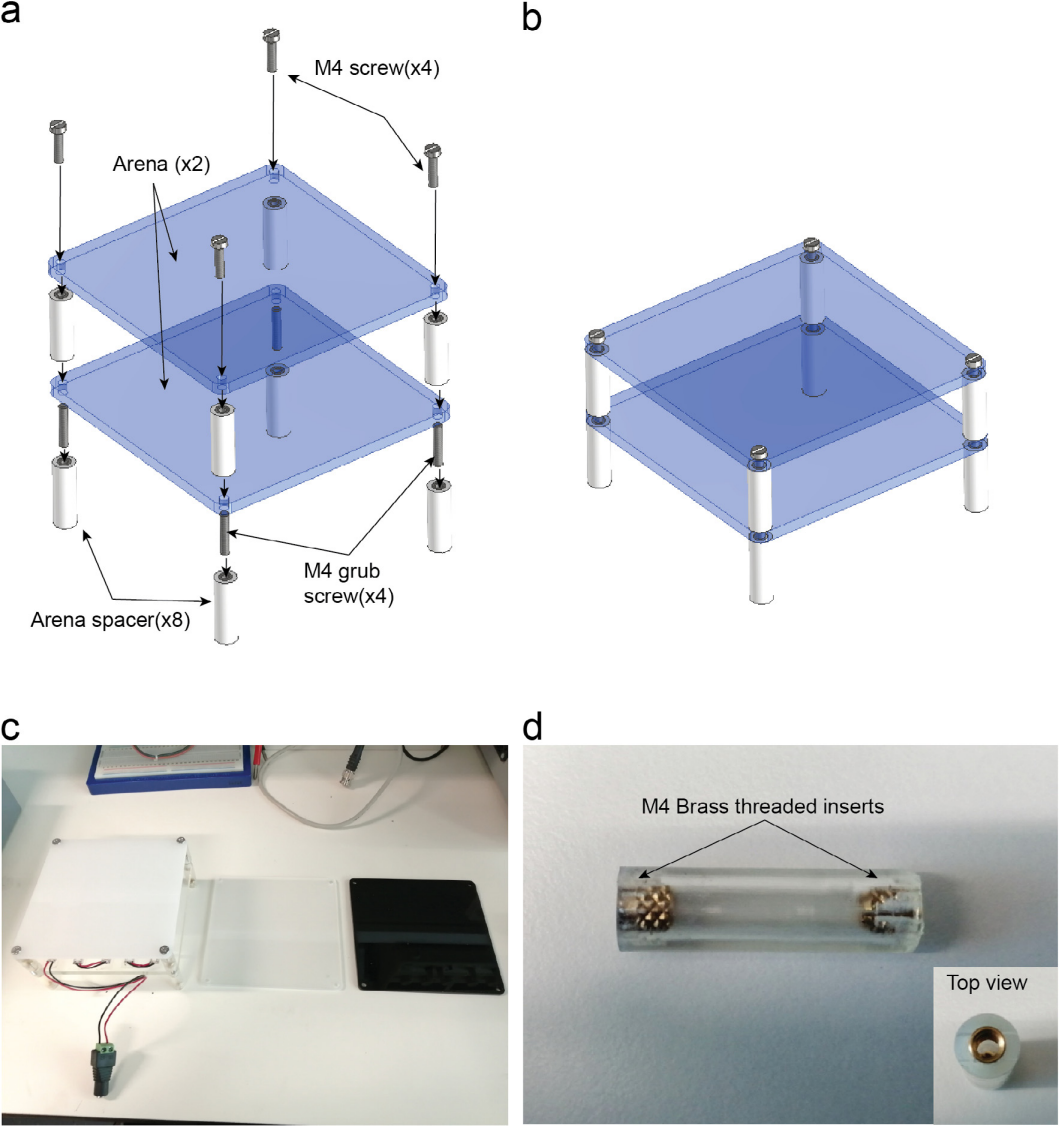
Arena assembly. a. Exploded view. b. Assembled view (Supplementary video 8). c. Different laser cut arena sheets using materials with different opacities. d. Lateral view of the spacer with threaded inserts and top view of the spacer.

Laser cut the Arena and 3D print the spacers. The arena can be laser cut with different materials with different optical properties to get diffusivity or opacity of the matrix placed underneath (Fig. 12c).

Insert in every 3D printed arena spacer M4 threaded inserts on each side (Fig. 12d). If you use FDM 3D printing they can be inserted heating them with the soldering iron.

### 5.6 Cart assembly

**Fig. 13:**
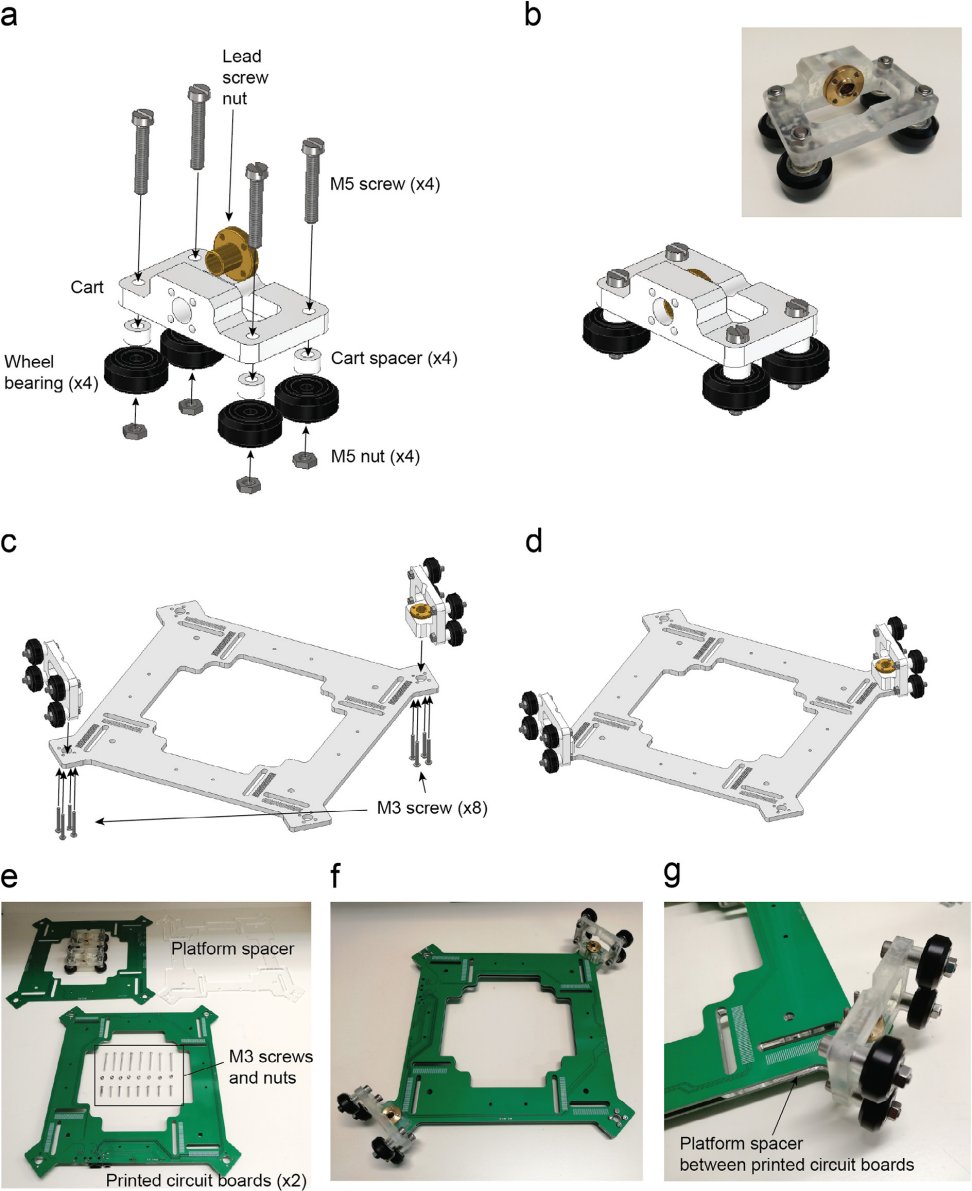
Cart assembly. a. Exploded view. b. Assembled view (Supplementary video 9). Platform assembly. c. Exploded view of the platform assembly. d. Platform assembled with both carts (Supplementary video 10). e. Materials needed to assemble the carts and the platform, Long M3 screws (40 mm) are needed to hold the nut in place. f. Assembled platform with two carts. g. Detail of the platform spacer laser cut in PMMA between two printed circuit boards.

Attach the wheels with the cart placing a cart spacer in between and a M5 screw in between (Fig. 13a-b).

Insert the lead screw nut on the central hole of the cart (Fig. 13a-b).

Attach two carts with the platform using four long M3 screws. The screws are attached to the lead screw nut (Fig. 13c-d).

The platform is composed by two printed circuit boards that are assembled on top and bottom of the platform spacer laser cut using PMMA (Fig. 13e-g).

### 5.7 Platform assembly

**Fig. 14:**
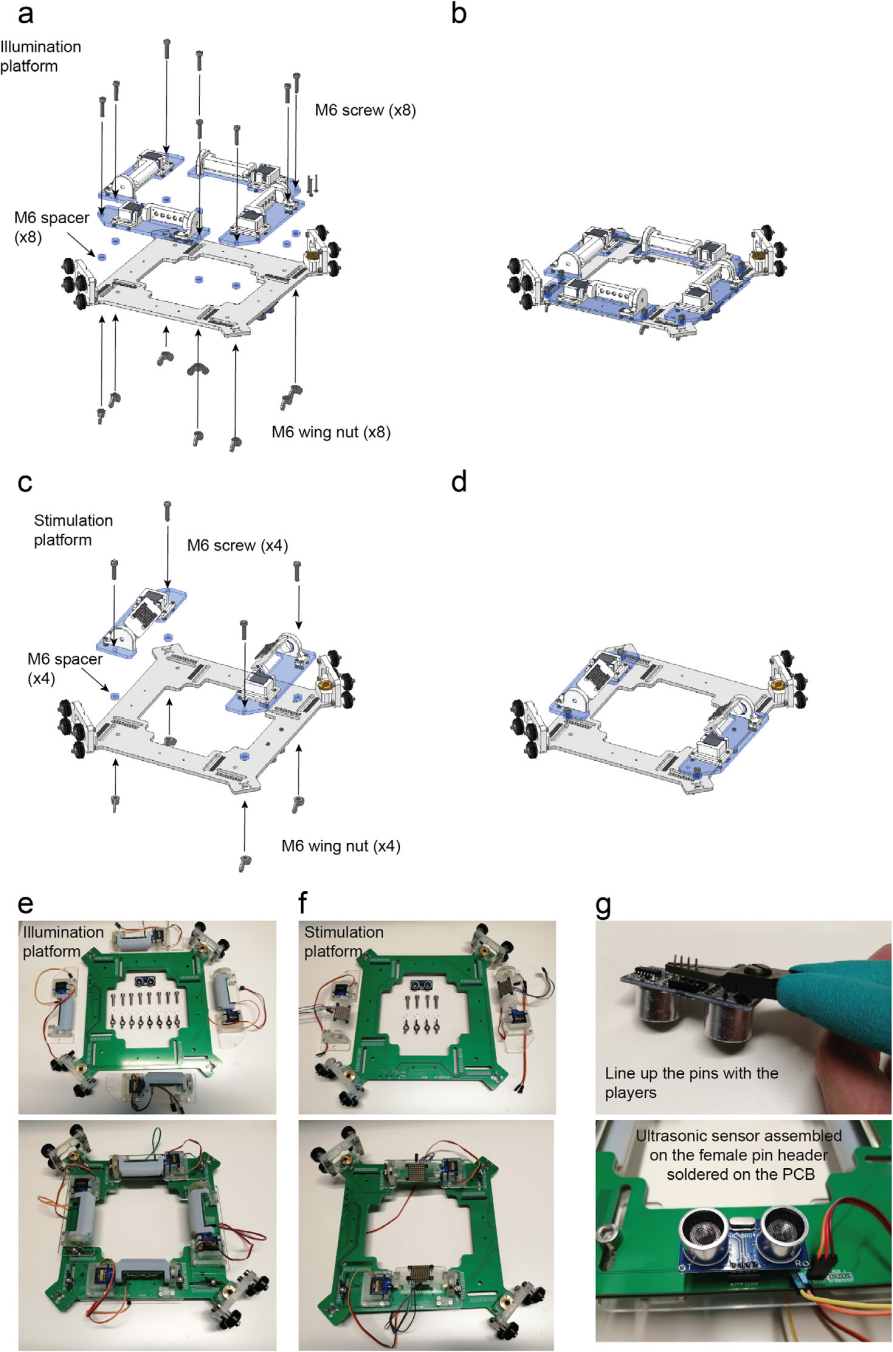
Platform assembly. With IR light sources. a. Exploded view. b. Assembled view (Supplementary video 11). Platform with RGB light sources assembly. c. Exploded view. d. Assembled view (Supplementary video 12). e. IR light sources assembled with the platform. f. RGB light sources assembled with the platform. g. Ultrasonic sensor assembly.

Using M6 screws, M6 spacers and M6 nuts the four IR light sources previously assembled can be now attached to the platform (Fig. 14a-b and Fig. 14e).

The same applies for the two RGB matrices (Fig. 14c-d and Fig. 14f).

The Ultrasonic sensor can be directly connected on the PCB female pin headers (Fig. 14g).

### 5.8 Device final mechanical assembly

**Fig. 15:**
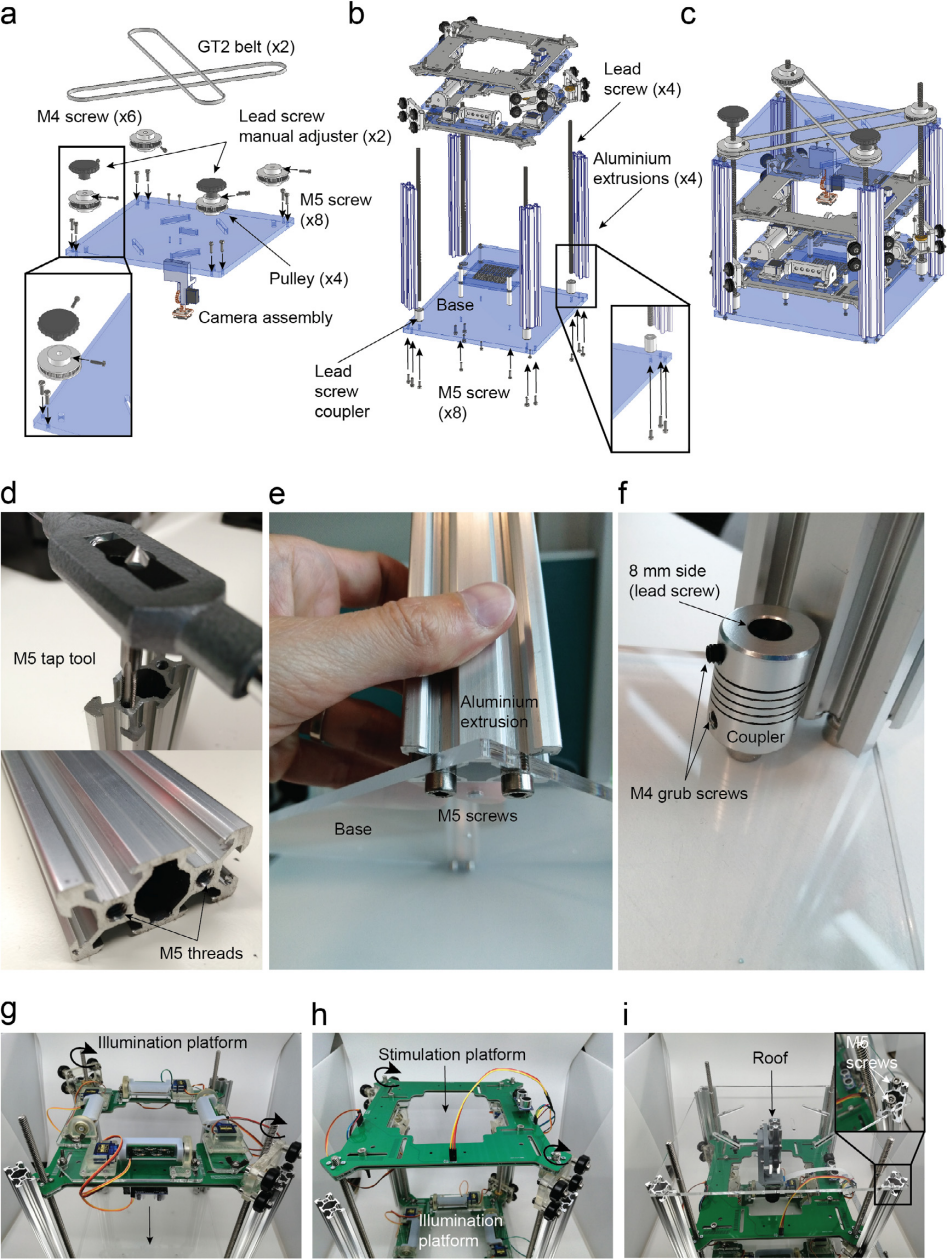
Structure assembly. a. Belt system assembly. b. Assembly of aluminium extrusions 2040, lead screws, arena and both illumination platforms. c. Device assembled (Supplementary video 14, 15, 16). Aluminium extrusions and lead screw coupler details. d. Tap tool M5 size and M5 treads after the process. e. The aluminium 2040 v-slot extrusions are attached with M5 screws to the base after threading the holes they have on both sides. f. The lead screw coupler is held by a M5 screw placed through the base. Assembly of the main structure. g. Illumination platform introduced by the top. h. Stimulation platform introduced by the top. i. Roof installation using M5 screws.

Use two M3 screws to attach the camera stage with the roof (Fig. 15a).

Attach the roof with the structure using eight more M5 screws (Fig. 15a).

Put the four pulleys on their respective lead screws keeping one diagonal higher than the other one, at least the thickness of the belt (6 mm). Use an M4 grub screw for each pulley (Fig. 15a).

Attach the arena to the base using four M4 screws (Fig. 15b).

The lead screws can be now inserted to the four couplers and tightened using the radial M4 grub screws of the coupler (Fig. 15b).

The two platforms can be inserted by rotating manually the lead screws and making sure that the platform does not tilt (Fig. 15b).

The four aluminium profile holes have to be threaded with an M5 tap tool (RS Components 917–2599) (Fig. 15d).

Then the aluminium extrusions can be attached to the base with M5 screws (Fig. 15e).

The couplers can also be attached to the base using M5 screws (Fig. 15f).

Both illumination first and stimulation after are introduced by the top sliding the cart wheels along the aluminium extrusions while simultaneously screwing the lead screws in clockwise rotation (Fig. 15g – 15 h).

Introduce the roof from the top aligning the four holes with the lead screws and inserting two M5 screws on each corner (Fig. 15i).

**Fig. 16:**
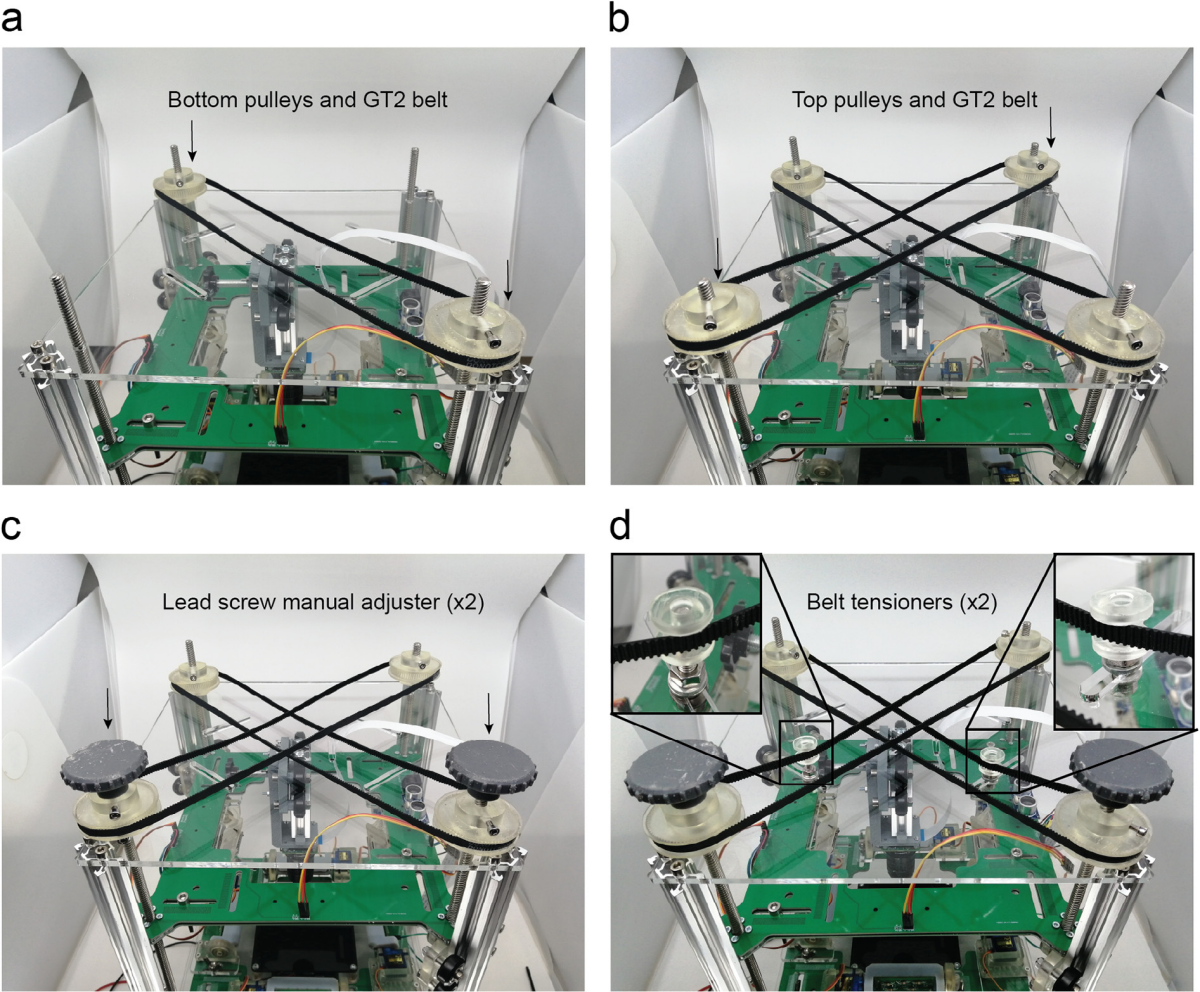
Timing belts assembly. a. First pair of pulleys. b. Second pair of pulleys. c. Manual adjusters. d. Belt tensioners.

Insert the first pair of pulleys with its belt without tension and tighten the screws against the lead screws (Fig. 16a).

Insert the second pair of pulleys with its belt without tension and tighten the screws against the lead screws in a higher position leaving space for both belts (Fig. 16b).

Insert and tighten the manual adjusters (Fig. 16c).

Use the guides on the roof to create tension on the belts using and M6 screw with the belt tensioner (Fig. 16d).

### 5.9 Soldering

**Fig. 17:**
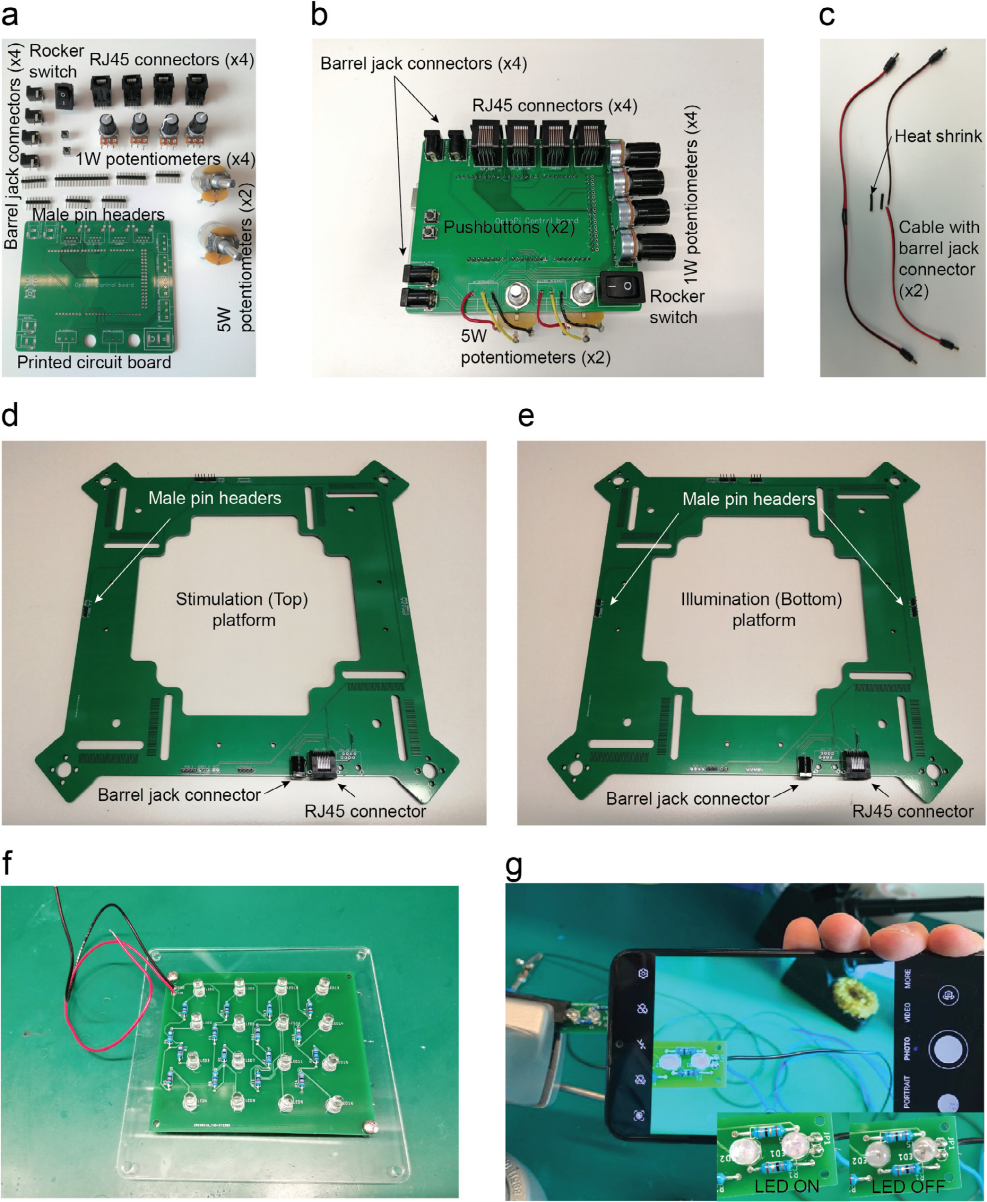
Control board shield soldering. a. Components required for soldering the control board. b. Control board with its components soldered. c. Platform power supply cables soldering. d. Stimulation platform with soldered components. e. Illumination platform with soldered components. f. Detail of the cable connections and colour codes. g. The phone camera can be used to evaluate if the IR LED are on when we apply power (5 V DC).

Solder the RJ45, barrel jack, two 5 W potentiometers, one 1 W potentiometers and male pin headers on the control board, rocker switch (Fig. 17a-b).

Solder the two power supply cables with the barrel jack connector using a heat shrink to protect the two lines from undesired contacts (Fig. 17c).

Solder the ethernet connectors, barrel jack and male pin headers on the stimulation platform on the positions DC1, Out_top, Servo 1, Servo 3, RGB 1 and 2 on the side without silkscreen. Solder Female headers on the Ultrasonic_T on the silkscreen side (Fig. 17d).

Solder the ethernet connectors, barrel jack and male pin headers on the illumination platform on the positions DC1, Out_bottom, Servo 1, Servo 2, Servo 3, Servo 4, IR1, IR2, IR3, IR4, and 2 on the side without silkscreen. Solder Female headers on the Ultrasonic_B on the silkscreen side (Fig. 17e).

Solder the 82 Ω resistors, power supply cables and IR LEDs on the matrix (Fig. 17f).

Check if the LEDs are working. You can use your mobile camera to catch the infrared light (Fig. 17g).

### 5.10 Electrical connections

**Fig. 18:**
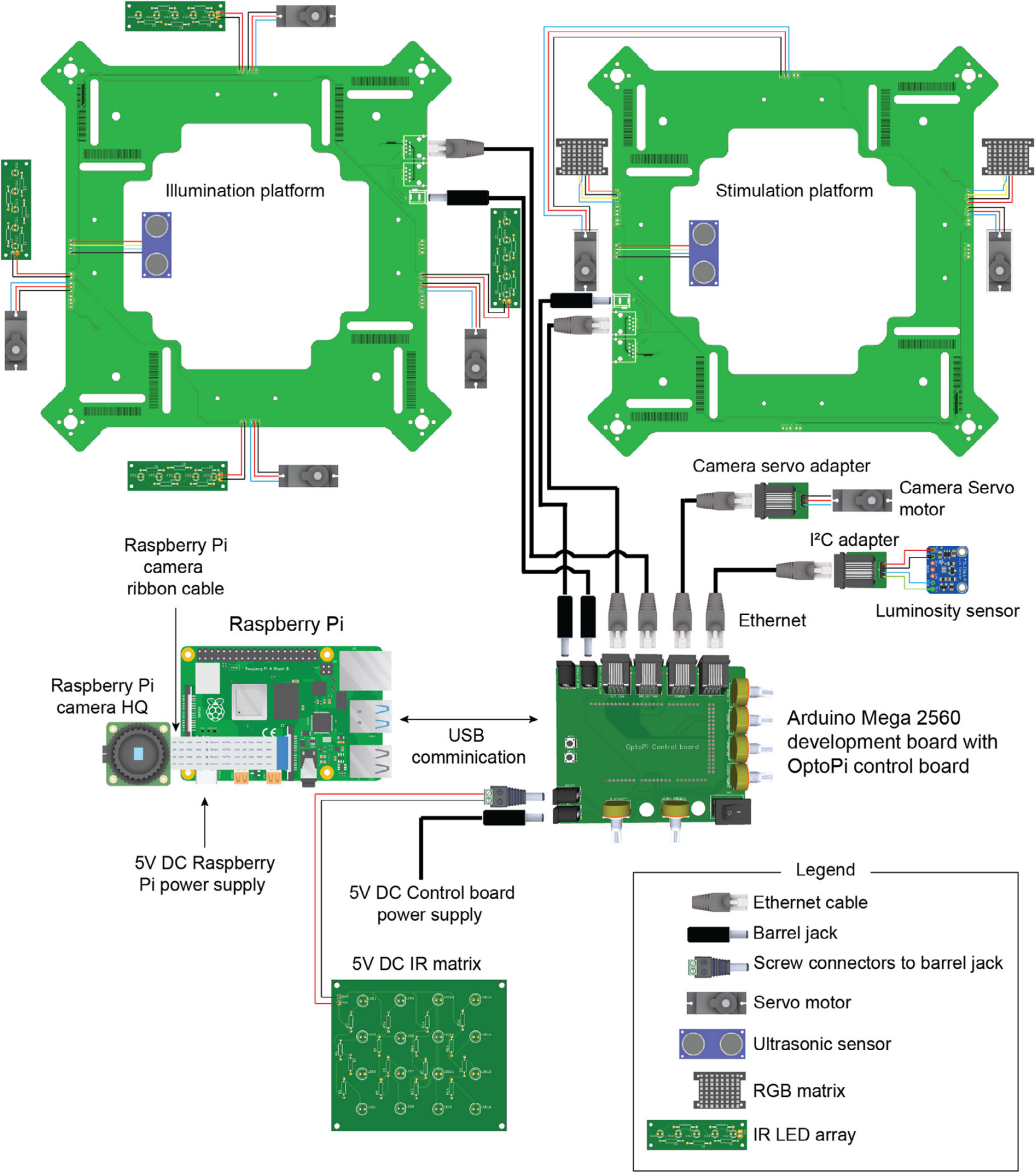
Electrical connections. The wiring required for all the actuators, sensors and illumination with the main control board.

**Fig. 19:**
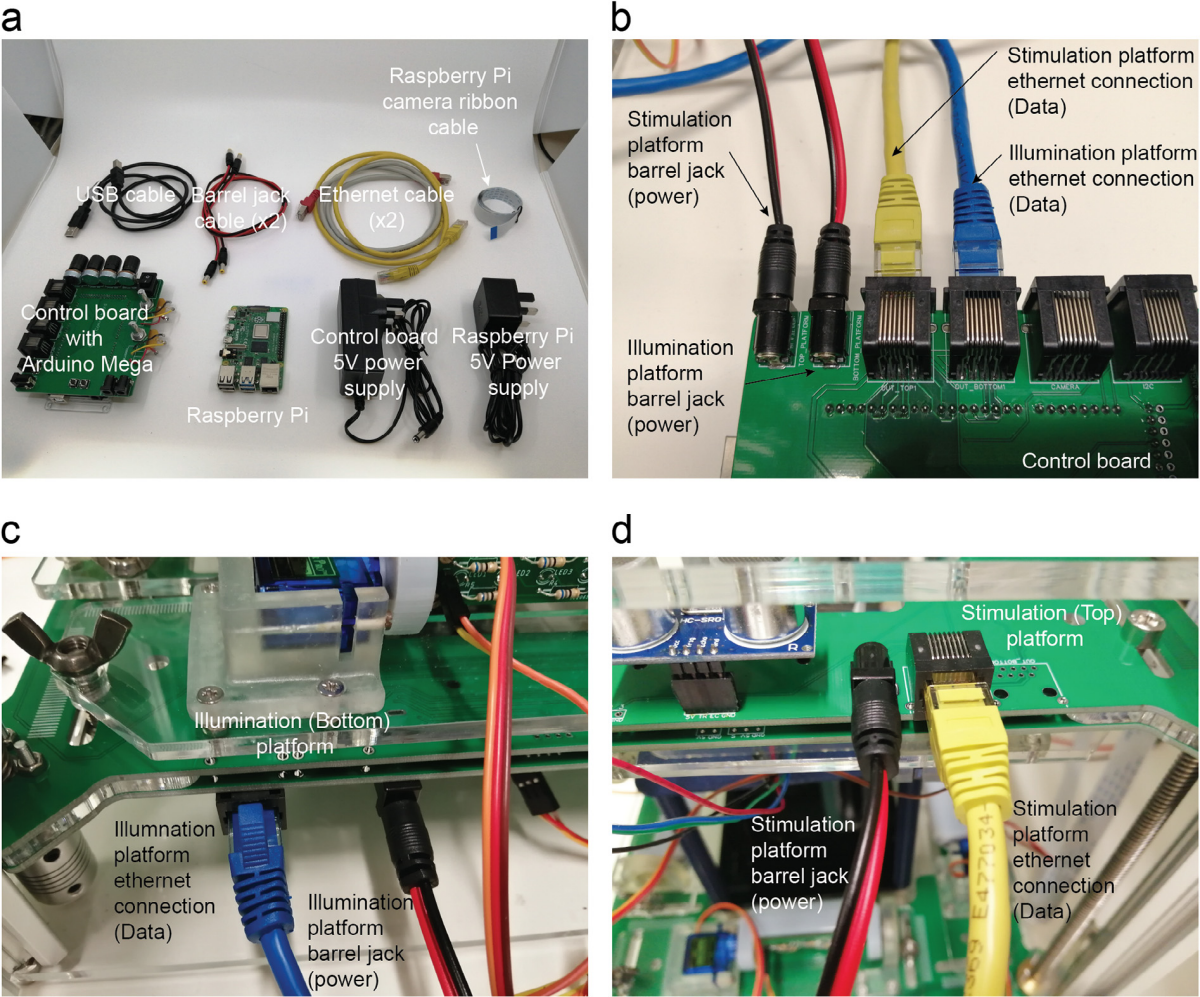
Main connections of the control board. a. Boards and wires required for the final installation and wiring of the OptoPi. b. Data and power connections on the control board. c. Connections on the illumination platform. d. Connections on the stimulation platform.

The electrical connections are described in Fig. 18. All the cables have been integrated on the platform PCB leaving only a power and a data cable that goes from the control board to each of the platforms. It has been designed like this to make the assembly easy and reduce soldering work and potential issues (Fig. 19a-d). There are two small PCB to adapt the I^2^C sensor breakout boards and the camera servo motor connector into an ethernet cable. The pi camera is connected to the raspberry pi using the ribbon cable. The Arduino is then connected to the raspberry pi. Finally, connect the power supply to the PCB Jack connector. The raspberry pi must be connected to a keyboard and mouse using the USB ports and a computer display using the HDMI port.

Most of the power required to operate the device comes from the illumination. The maximum current that the IR illumination can draw adding the four arrays is 1 A and increases up to 1.8A if you add the matrix illumination underneath the arena. Every RGB matrix operating at maximum brightness demands between 1 and 1.2 A depending on the colour. The Dotstar matrices can go to 2.5 A combining the three RGB channels (White). A high demand of light intensity requires high currents, if that is the case it is important to supervise the device.

### 5.11 Ease of manufacturing

The device is composed by several moving parts, some of them are 3D printed and some of them are cut with a laser cutter. The laser cutting process can take approximately one hour in a CO_2_ laser cutter with 600 × 300 mm cutting bed area. The printing process of all the parts using a Formlabs Form 3 resin 3D printer will require filling seven building beds with prints that will have from four to six hours duration. The assembly of the mechanical components will take approximately 20 h. The Printed circuit boards have through hole components making it very easy for an unexperienced user to finish the soldering process in less than 4 h. The electrical connections can be done very quickly by just connecting Power supply and ethernet cables for each platform and in that way eliminating tedious wire soldering of each individual component.

## 6 Operation instructions

This section has the following subsections:

6.1. Programming the Arduino Mega 2560 in Windows systems.

6.2. Relationship of functions in the Arduino code.

6.3. Acquiring video recordings from the Raspberry Pi.

6.4. Reproducing OptoPi settings.

6.5. Analysing videos with BIO.

6.6. Running BIO in on-line mode.

### 6.1 Programming the Arduino Mega 2560 in Windows systems.

Download and install Arduino environment on the computer (https://www.arduino.org) and install the following libraries:

- Adafruit GFX.

- Adafruit DotStarMatrix.

- Adafruit DotStar.

- Adafruit sensor.

- Adafruit TSL2561_U.

- Adafruit AS7341.

- Elapsedmillis.

The other required libraries SPI, Wire (I2C) and servo are included in the standard libraries of the Arduino IDE.

Open the Arduino script: OptoPi.ino.

- From the ‘‘Tools” tab:

- Select from ‘‘Boards” the ‘‘Arduino Mega or Mega 2560″.

- Select from ‘‘Processor” the ‘‘ATmega2560″.

- Select from ‘‘Port” the ‘‘COM (Arduino Mega or Mega 2560)”.

- Compile and upload the code (clicking on the sideways arrow).

The arena is ready to be used.

### 6.2 Relationship of functions in the Arduino code:

The main functions can be divided in two main groups: setting functions and automated functions.

The setting functions enable the control of the device elements using the knobs and interfaces. This mode of operation is also used to read the values of the sensors by opening the serial port (Tools -> Serial monitor). These functions can be set-up once and the device can then be used by just using the knobs. This is the mode of operation for simple recording experiments, or to find the right settings for a future automated experiment.

The automated functions are those that enable using the device with automatic experimental protocols that actuate the device without the user intervention. For example, it can create an optogenetic blinking series - *blink()* function - or an intensity ramp – *intensityramp()* function -. The parameters that receive the functions to operate are global variables declared at the top of the program. After the *setup()* function that is always executed once, on the main *loop()* function, the program waits for the user to press “Enter” key from the keyboard connected to the Raspberry pi. The values printed are read by the Raspberry Pi and saved into a txt file with timestamps.

Setting functions.

The setting functions are functions intended to be used to adjust parameters of the setup or simply control the setup manually without any protocol automation.

*void* Settings ().

This function prints on the serial port the distances of the two ultrasonic sensors (Top and bottom), the camera distance, the angle of the two light sources (IR and RGB) and sets the RBG matrices brightness and at the same time is reading the knob values so the user can control all those parameters and adjust them.

*void* read_irradiance ().

This function reads the value sent by the TSL2561 and converts the illuminance into irradiance using the calibration equations. Depending on which of the boolean variables red, green, blue are set to true the specific calibration equation will be choosen.

*void* read_spectral_sensor ().

It reads the incoming data sent through the I^2^C bus and prints on the serial port the normalized value for each wavelength from 415 nm to NIR (optional if connecting the Adafruit AS7431 module).

*void* Set_Matrix_Color_Brightness (*byte* Red_int, *byte* Green_int, *byte* Blue_int, *byte* brightness).

The function receives four input parameters of the byte types, the first three are variables that can be found at the beginning of the program, they correspond to the value from 0 to 255 of red, green and blue in that order. The fourth byte is the brightness also from 0 to 255.

Automated functions.

The automated functions are required only if automatic optogenetic stimulation is required. If the user wants to control the setup manually, these functions are not required. Both (ramp and blinking) functions cannot be used at the same time as they control the RGB matrices in a different way.

*void* intensity_ramp (*byte* max_value, *unsigned int* interval).

**Fig. 20:**
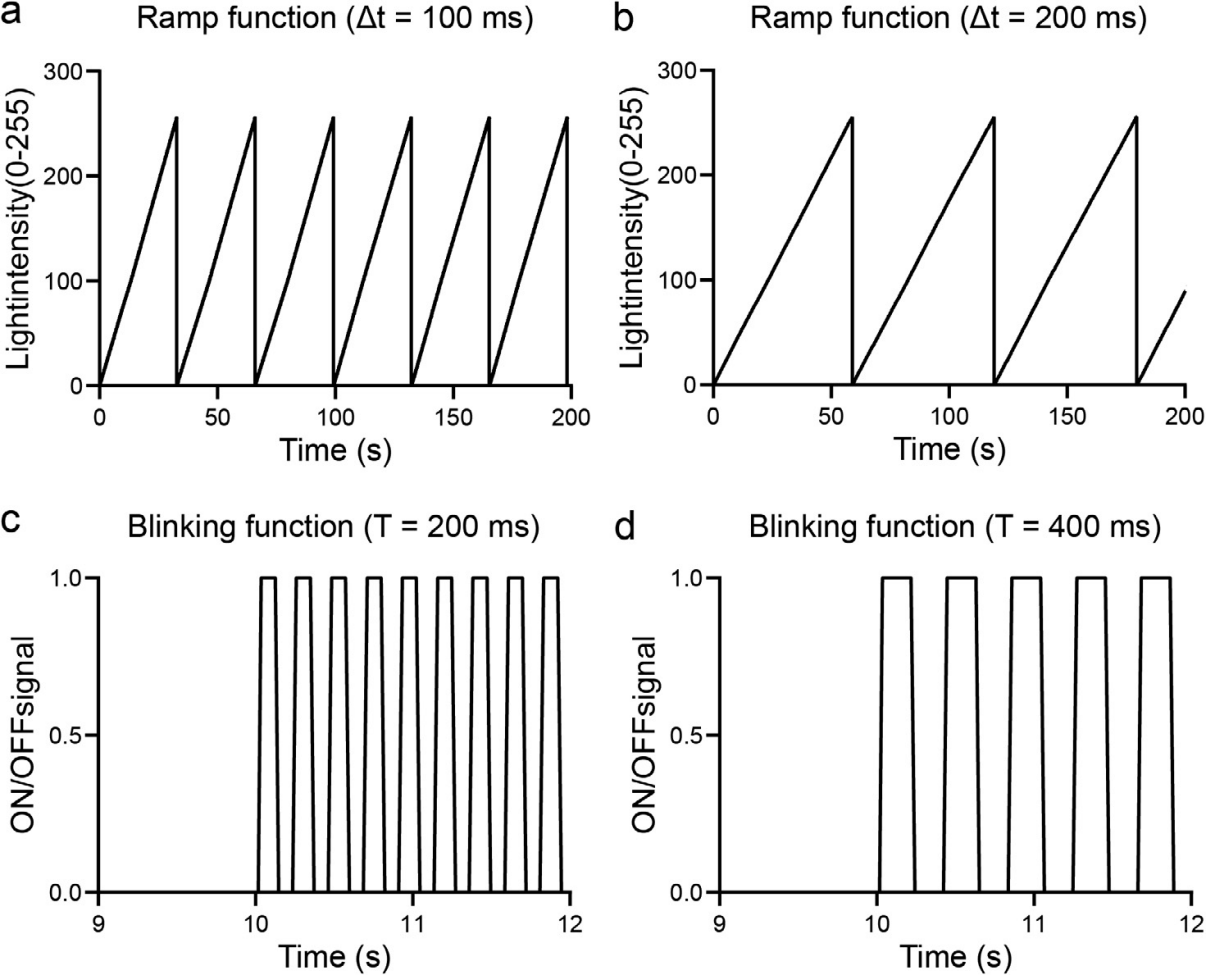
Automated functions. a. Ramp profile of light intensity, created by increments of one byte spaced 0.1 s. This pattern is generated by the automated function intensity_ramp(). b. The same function called with 0.2 s interval. c. ON/OFF cycles generated with both RGB matrices with a period of time of 0.1 s between ON and OFF states and an initial time of 10 s in off position. This pattern is generated by the automated function blinking. d. The blinking function using a delay of 0.2 s.

The ramp function increases linearly the value of the light intensity up to a maximum value (max_value) given a certain interval which is defined at the beginning of the program as DELAY (Fig. 20a-b).

*void* blinking (*unsigned int* interval_low, *unsigned int* interval).

The blinking function generates ON/OFF cycles with a fixed light intensity and an initial waiting period defined as DELAY_0 at the beginning of the program. In this case the variable DELAY is also used as interval between the ON and the OFF cycles (Fig. 20c-d).

*void* Blink_from_Pi_serial().

The Blink from Pi serial sets the Arduino Mega to be constantly reading from the serial port. It expects to receive “0″ or “1” from the raspberry Pi to put the RGB matrices either HIGH or LOW state.

*void* Custom_intensity_from_Pi_serial().

This function is also constantly reading the serial port and using the value sent from the Raspberry Pi to control the RGB light intensity within the range of 0–255.

### 6.3 Acquiring video recordings from the Raspberry Pi.

Videos in this manuscript have been acquired through two methods: 1) using the Raspberry Pi 4 via a Python script (‘Camera_acquisition.py’) operating on a Raspbian OS, and 2) directly via BIO on an Ubuntu OS (see ‘Analysing videos with BIO' below).

The ‘Camera_acquisition.py’ communicates with the Pi camera module to enable the user to modify video acquisition parameters including framerate, frame resolution and frame colour. As such, the 'Camera_acquisition.py’ script can be easily modified to meet a user’s needs. To simply record a video, lines 8, 21–28, 39, 41 and 45–51 can be commented out. However, should the Arduino’s ‘state’ need to be recorded in addition to a video, for instance, if an optogenetic stimulus is being delivered, keep the ‘Camera_acquisition.py’ script unedited. For synchronisation between the Arduino and the Raspberry Pi first upload the Arduino script (‘OptoPi.ino’), commenting out the desired protocol, for instance, *settings()* or *blinking()*. Having ensured that the OptoPi.ino script has uploaded correctly (receiving a prompt from the Arduino IDE) simply run the ‘Camera_acquisition.py’ script via your chosen Python IDE. This will prompt the user with an unrecorded preview enabling the user to make any modifications before video acquisition begins. The user is prompted to press the ‘Enter’ key to begin recording and upon doing so the Raspberry Pi will send a serial command to the Arduino. As currently written, when the ‘OptoPi.ino’ is uploaded to the Arduino, the Arduino is essentially kept in an inactive state until prompted by a serial command. Therefore, upon pressing the ‘Enter’ key, the Raspberry Pi sends this serial command thereby activating the Arduino to begin the selected protocol. The Raspberry Pi will next create a start time point and begin recording the video. As a part of enacting its selected protocol, the Arduino will send via serial port a string detailing its current ‘state’, for example, LED matrix intensity. This information is decoded by the Raspberry Pi and written to a.csv file along with a timestamp thereby providing information as a series of timestamps as to when stimuli are being delivered by the Arduino. This process is repeated with the Raspberry Pi assessing whether the time elapsed exceeds a user defined experiment duration (the variable ‘experiment_duration’). Once the required time has elapsed the Raspberry Pi ceases recording the video. As a part of debugging, a user may need to upload their ‘OptoPi.ino’ several times prior to running the ‘Camera_acquisition.py’ script to ensure that the Arduino is in an ‘inactive’ state and awaiting the appropriate serial command from the Raspberry Pi.

The steps to take are the following:

1. Install the required python libraries, these are as follows:

- Pyserial.

- Picamera.

- Time.

- System.

- Datetime.

The only one you will probably have to install is the pyserial library. But first you must ensure you have python3 installed by typing the following command in the terminal:

python 3 – version.

Should this return a value, that means you have correctly installed a version of python3 and may proceed. Otherwise, type the following commands:

sudo apt-get install python3.8 python3-pip.

sudo apt install python3-pip.

Then you install the pySerial library by typing.

python3 -m pip install pyserial.

2. Open the terminal and write: ls /dev/tty* and press enter. This will open the list serial ports available. If you do that before and after connecting the Arduino board, the name that only appears when the Arduino is plugged is the name of the Arduino serial port usually ‘/dev/ttyACM0′, ‘/dev/ttyUSB0′, or similar. Alternatively, copy the name of the port the Arduino is connected to via the Arduino IDE by clicking the dropdown ‘Tools/Ports’ and find your connected Arduino.

3. Copy the “Camera_aquisition.py” inside a folder on the Raspberry Pi desktop and open the script in “Camera_aquisition.py” via Thonny, a Python IDE already available on Raspbian, or any other IDE of your choice.

4. In the script modify the “Acquisition_time” and write a “file_name” of choice and press RUN. A window with the video recording will open and a.CSV file with the name you assigned will be automatically created and the timestamps of data sent from the Arduino code will be logged in for the duration of the acquisition.

### 6.4 Reproducing OptoPi settings

The device can report quantitative information about the position of its elements while adjusting the experimental conditions. The function *Settings()* reports the angle of the IR and RGB servo motors, the position of the camera stage and the distance measured from the ultrasonic sensors as the vertical positions of both platforms. When you are running the *Settings()* function on the Arduino main loop, all the servo motors can be controlled using potentiometers as well as illumination/stimulation brightness. The information is constantly sent through the serial port and it can be saved on the Raspberry Pi csv either to keep track of the experimental settings or share these conditions with other colleagues.

The steps we recommend are:

 Upload the Arduino sketch with the Settings() function as the only uncommented line in the loop() function.

 void loop()

 {

 String command = Serial.readString();

  while (command == “begin”)

  {

  //Setting functions

  Settings();

  }

 }

In the raspberry pi python script: video_annotations_sync.py, change the name of the exp variable in line 11 for ‘Settings’, this will export the settings reported by the Arduino every loop cycle in a single line labelled as: Distance bottom (Db), Distance Top (Dt), IR illumination angle (IR), RGB angle (RGB), Camera distance (Cd), RGB brightness (Bright).

In the same way as described on the device operation, once you run the python script on the Raspberry Pi, it will wait until you press the enter key and then it will start receiving data from the Arduino settings and save this data on the CSV file in the format previously described.

In addition, this can be interesting for some users where the experimental conditions don’t require the optogenetic stimulation and they only want to adjust settings manually while doing the experiments.

### 6.5 Analysing videos with BIO

Once the video has been acquired it can be analysed using multiple software. Here we present BIO, a multi-animal tracker that is particularly useful for on line real-time tracking. Importantly, because it is lightweight is compatible with low-cost hardware such as those used for OptoPi.BIO has been tested and works across operating systems including Windows and Ubuntu (Ubuntu Desktop 21.04 (RPi 4/400) on Raspberry Pi). For Windows systems, the latest BIO binary can be downloaded and installed from *https://joostdefolter.info/bio-research*. For Ubuntu, BIO can be built following the instructions outlined in installation notes found here *https://github.com/folterj/BioImageOperation*.

This code repository also includes a description of the steps required to generate a tracking script, the functions available within BIO, and a number of example scripts for different use cases. As a starting point, a template script can be generated by selecting ‘Generate tracking script’ in the menu and subsequently selecting a video file to be analysed. Once this has been generated, the user can follow the steps laid out in this script to first create a background image, assign appropriate thresholds to properly track the objects of interest, and finally output these tracks as data output and/or video. Initially, the user can run BIO instructions without specifying parameters. BIO will then estimate parameter values, which can be shown and subsequently fine-tuned for optimal performance. BIO also allows showing intermediate image output at any stage which also aids this process providing the user insights into each step of the script.

Example scripts used to track videos are provided, but a brief description of how they were tracked is as follows. Videos, image sets or live capture are used to acquire images. Images are converted to grayscale for the following steps. A background image can be generated with these steps as well. Provided all the potential subjects of interest moved during the video you will be left with a background only including the static objects present in the video. For single images we recommend the use of a lossless compression image format, such as PNG and TIFF. It is this background image that potential objects are compared against by using a subtract function. The precise threshold, which is a percentage that any object of interest must differ to the background, is user defined and is a decimal value from 0 to 1. The higher the contrast of the source images, the more stringent the threshold (i.e. higher) can be while reducing sensitivity to image noise. For the example videos provided we used thresholds below 0.1.

An optional image mask can also be provided removing any areas in the source image that are not of interest. Pixels where the mask has a positive value will be kept while those where the mask has a value of 0 will be removed from the original image. Mask images are easily made using image tools such as GIMP (https://www.gimp.org) and imageJ/FIJI (https://imagej.net).

The final steps involve assigning clusters, to delineate which objects to track, and the actual tracking. Assign a minimum and maximum value to the cluster function for BIO to detect objects of interest. Finally, assign values to the tracking function to define the maximum acceptable movement per frame, the minimum number of frames a cluster is recognised as active, and finally the maximum number of frames a cluster is considered inactive. Once these settings have been optimised, the tracking data and/or images can be saved to file. Additionally BIO can overlay the source images with the clusters/tracks to visualise the tracking.

Note, that using a wide-eye lens to record animals in a wide arena will produce videos with barrel distortion, i.e. objects and distances in the centre of the arena appear larger than those towards the edges. For the experiments performed for this publication arena sizes were not large enough for such barrel distortions to become an issue. However, for accurate tracking in wider arenas it is essential to correct these distortions before tracking. Such corrections can be performed using BIO by first imaging a checkerboard pattern under the camera position conditions used for experiments and performing the optical calibration function on images and videos. Example checkerboard image with distortion, with distortion corrected and example scripts are available in Supplementary folder visual_distortion_correction.

### 6.6 Running BIO in on-line mode.

The steps required to run BIO online are the same as outlined above, but rather than selecting a single video, the user must use the capture function (‘*OpenCapture(0)*’). If BIO detects a functioning camera, it will read a live feed from it. When using the online mode, a background image can be computed using an image capture loop for a suitable period of time and calculate the median image, in the same script before the main capture/tracking loop. Additionally, BIO also supports a dynamic background using the update background function.

## 7 Validation and characterization

This section has the following subsections:

7.1. Experimental characterisation of the illumination and recording modules.

7.2. Technical characterisation of the optogenetic modules.

7.3. Technical characterisations of the precision of the device.

7.4. Timing precision testing.

7.5. Experimental characterisation of the optogenetic modules.

### 7.1 Experimental characterisation of the illumination and recording modules

**Fig. 21:**
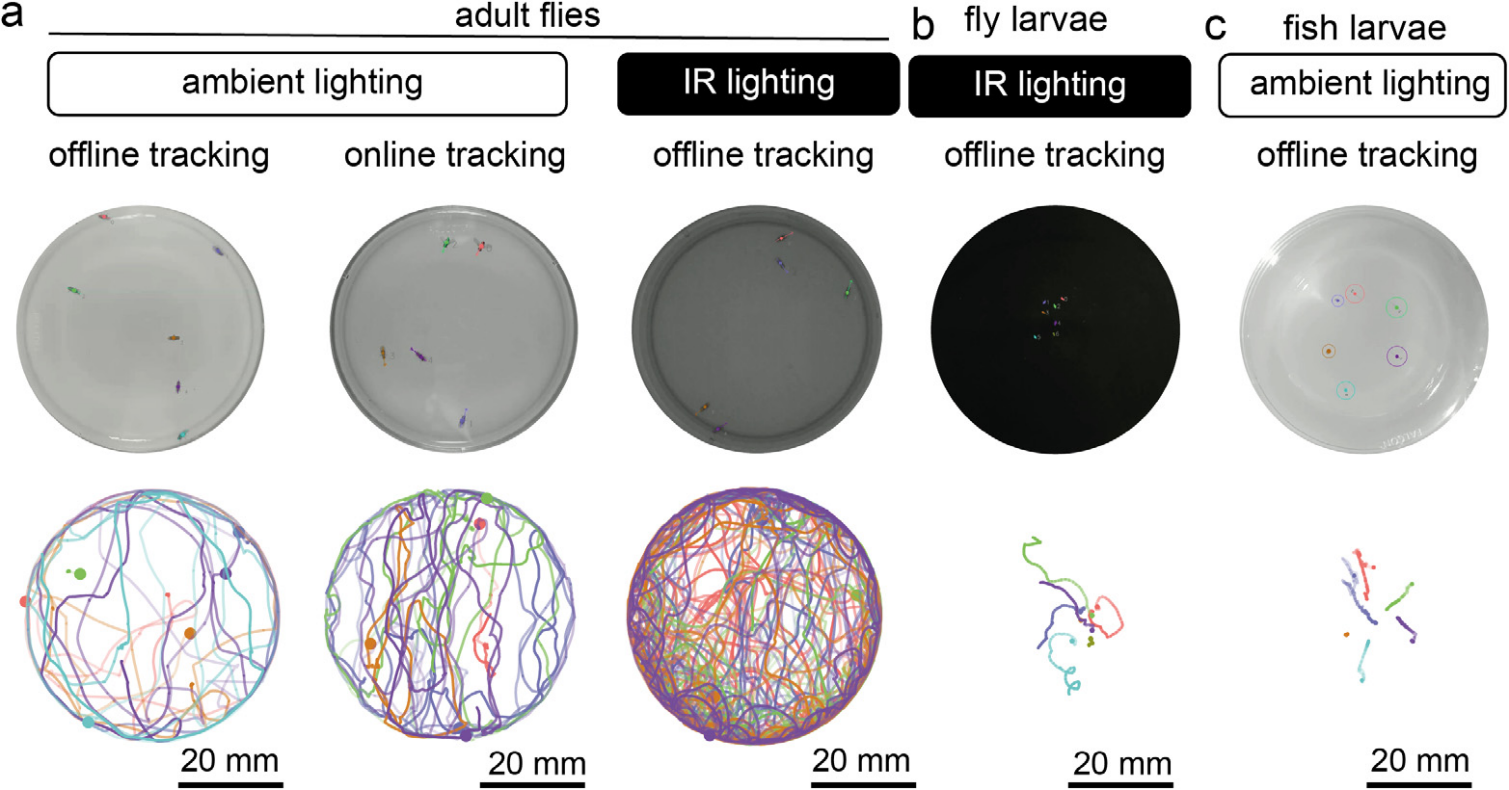
Experimental characterisation of the illumination and recording modules. a. Example *D. melanogaster* adult snapshots (upper panels) and trajectories (lower panels) under different lighting and recording conditions (left and middle under ambient lighting, right in infrared lighting, middle column is tracked live). b. Example *D. melanogaster* larvae free-roaming in infrared lighting, example image (upper panel), total trajectories (lower panel). c. Example free-roaming *D. rerio* larvae in ambient lighting showing example image (upper panel) and trajectories (lower panel).

To test the OptoPi’s performance, we used various lighting settings to record and analyse the behaviour of *Drosophila melanogaster* adult flies and 1^st^ instar larvae, as well as *Danio rerio* fish larvae (Fig. 21). We recorded animals in ‘ambient’ lighting conditions, wherein the overhead LED matrices emitted white light, and infrared lighting, either through darkfield illumination from the side IR LED matrices or backlighting from IR LEDs beneath the stage. We found that ambient lighting worked particularly well for adult flies and larval fish, as their dark pigmentation provided good contrast against the light background. However, the small and transparent body of 1^st^ instar fly larvae did not provide enough contrast under ambient light conditions, and these are best recorded using darkfield illumination from the side IR LEDs.

To demonstrate that each of these conditions enables animal tracking we analysed these experiments using BIO both online and offline (Fig. 21 and Supplementary Videos 17–24). Analysis and visualisation of larval trajectories were done in R relying on the package trajr (https://cran.r-project.org/web/packages/trajr/index.html).

### 7.2 Technical characterisation of the optogenetic modules

**Fig. 22:**
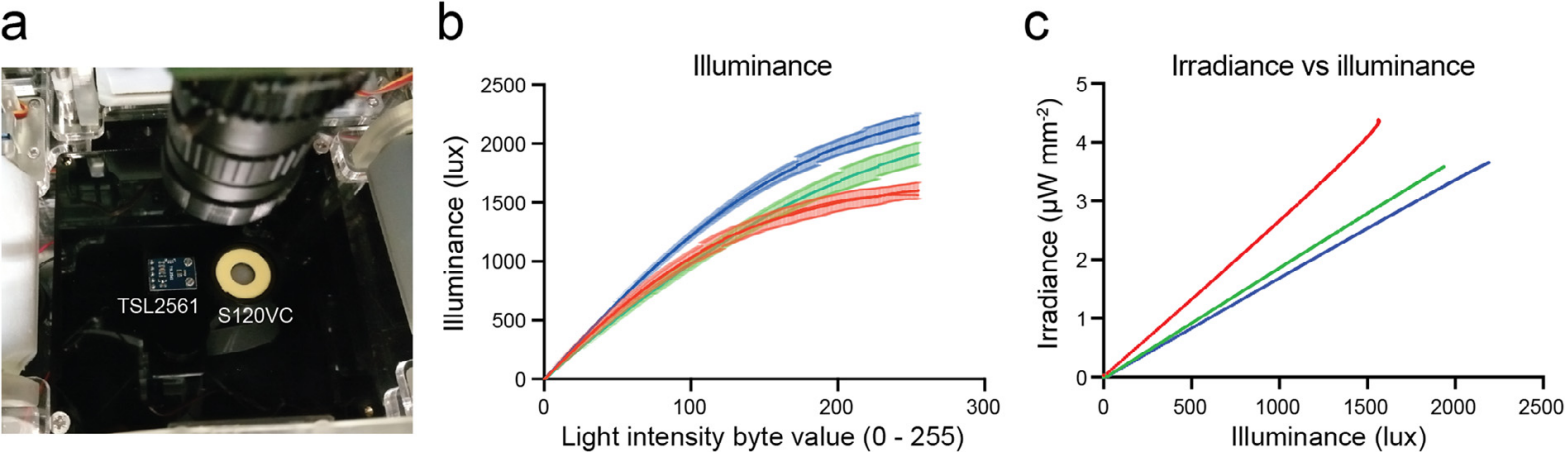
Technical characterisation of the optogenetic module. a. Irradiance characterization setup, the TSL2561 luminosity sensor (left) and the Thorlabs S120VC (Right) on the calibration arena. b. Illuminance functions obtained with two TSL2561 modules (Error bars correspond to the standard deviation). c. The calibration functions obtained comparing illuminance and irradiance functions red (633 nm), green (521 nm) and blue (465 nm) lights. (For interpretation of the references to colour in this figure legend, the reader is referred to the web version of this article.)

To characterize the capabilities of the optogenetic modules, the irradiance and illuminances were simultaneously measured on the set-up using a calibration platform with the in-built TSL2561 illuminance sensor and a calibrated Thorlabs S120VC photodiode placed on the centre of the arena (Fig. 22a). The Thorlabs S120VC was connected to a PM100D compact power meter console. The position of the two RGB matrices were at an angle of 100°, Bottom platform distance (2 cm) and distance of the top platform (11 cm). The experiment was replicated using two TSL2561 illuminance sensos. That position was selected as the maximum stimulation achievable with two RGB Adafruit DotStar high density matrices. Both sensor signals were obtained at the same time while performing an intensity ramp function (Δt = 1 s) with the RGB matrices (Fig. 22b). Then both functions (irradiance and illuminance) were fit in quadratic equations (R^2^ = 0.9887, 0.9911 and 0.9867 for red, green and blue curves) and the two functions were used to generate values to correlate the measurements of both sensors (Fig. 22c) with also high correlation coefficients (R^2^ = 0.9989, 1 and 0.9999 for red, green and blue functions). These equations have been introduced to the Arduino code inside the *read_irradiance()* function and can be used by the user to find the adequate position for the light stimulation. Depending on how the initial boolean variables for red, green, or blue are set, the function will convert the TSL2561 reading using the corresponding equation. These equations can be used to get an approximation of the irradiance on the arena for experimental and measuring purposes.

### 7.3 Technical characterisations of the precision of the device

The following table collects the main features of the device regarding the resolution of the different systems that measure the angular, horizontal, and vertical positions of the light sources as well as the camera. The maximum irradiance measured for red, green and blue light and their resolution, and the measured wavelengths for the optogenetic stimulation light sources and for the infrared illumination.

**Table:** 

**Parameter**	**Value**	**Unit**
Light sources angular resolution	1	°
Light sources horizontal resolution	1	mm
Platform vertical displacement resolution	10	mm
Camera stage linear resolution	0.2	mm
Light stimulation maximum irradiance (Red, Green, Blue)	4.45, 3.58, 3.69	µW/mm^2^
Light stimulation Irradiance resolution (Red, Green, Blue)	0.015, 0.013, 0.012	µW/mm^2^
Light stimulation wavelength (Red, Green, Blue)	633,521,465	nm
Illumination wavelength (Infrared)	940	nm

### 7.4 Timing precision testing

**Fig. 23:**
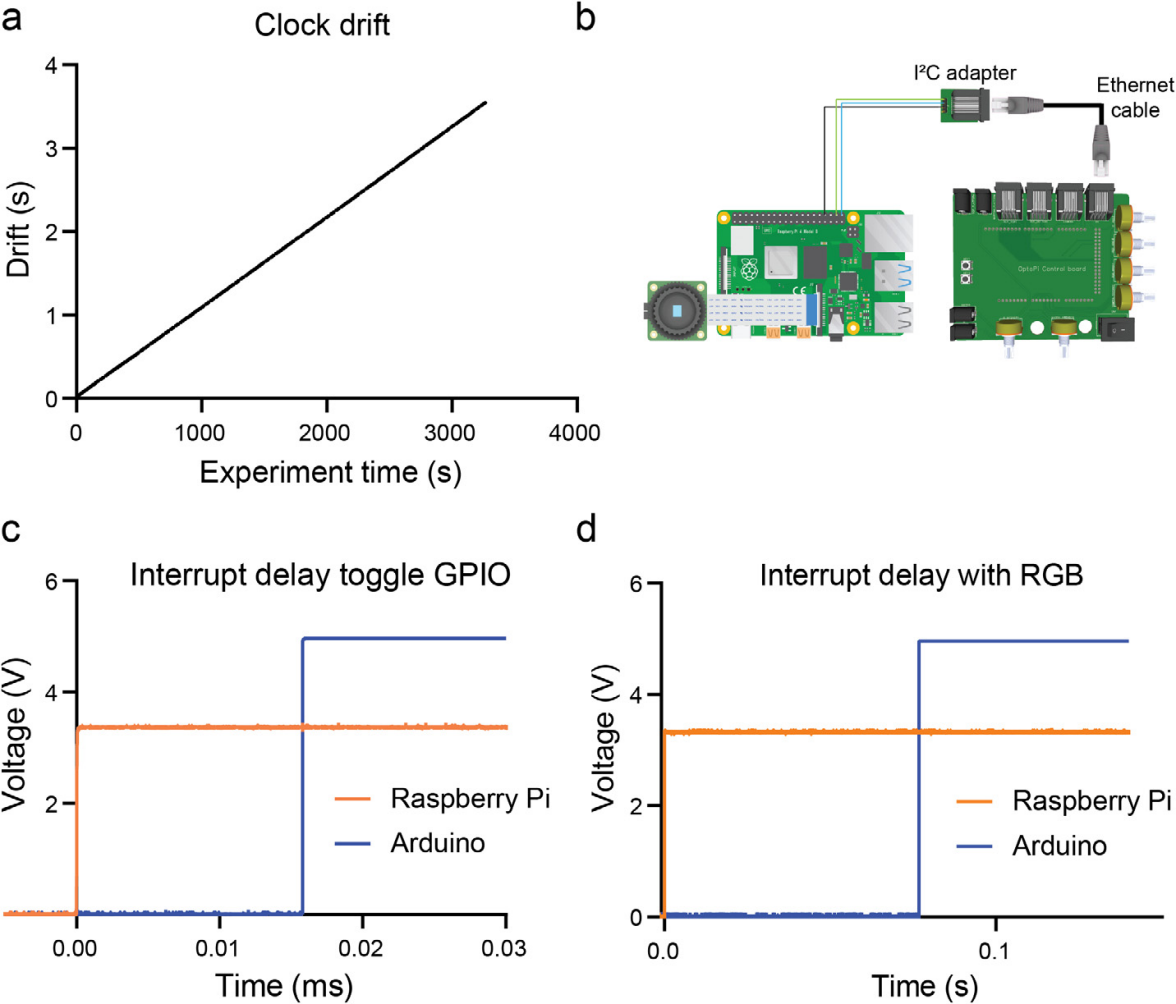
Time precision testing. a. Clock drift between the Raspberry Pi and the Arduino Mega 2560. b. Schematics of connections to connect GPIO 20 and 21 from the Raspberry Pi with the Arduino Digital Pins 20 and 21 (SDA and SCL) which have interrupt capability. c. Time elapsed between the change of the GPIO pin state of the Raspberry Pi and the change of state on the output digital pin of the Arduino, that changes with the interrupt routine. d. Time elapsed between the change of the GPIO pin state of the Raspberry Pi and the change of state on the output digital pin of the Arduino, that changes with the interrupt routine but in this case sending the command to actuate both RGB matrices.

The device was originally conceived to provide highly customizable illumination for automatic tracking of multiple small animals using the software package BIO. Given the number of degrees of actuation and illumination sources, we choose a widely available development board with a high number of digital and analogue pins. Subsequently, we though that an interesting feature would be to add serial communication between the Raspberry Pi (Image acquisition unit) and the Arduino Mega (Control unit) to enable automatic light stimulation protocols. In the standard configuration of the device, using the functions *intensity_ramp* and *blinking,* the Arduino keeps track of the stimulation times selected in its firmware. In this scenario, the Pi and the Arduino each have their own independent clocks, which are not synchronized. This leads to a temporal drift of approximately 3.96 s every hour (Fig. 23a). For the type of experiments, we were interested in performing (see Experimental characterisation below), this drift was not an issue. Additionally, in this configuration the Arduino is constantly (every 100 ms) reporting the value of the illumination to the Raspberry Pi over the serial port, and the Raspberry Pi stamps that value with its corresponding time and video frame. If the device is used under this configuration for long recordings, the researcher will experience a drift of the interval over time but the light stimulation value will be stamped with the Raspberry Pi time stamp corresponding when the data point was received, which means that this drift can be constantly monitored and corrected for during the analysis.

However, some users might want to not have to deal with this drift at all. We have explored two other possible configurations where the Raspberry Pi does not rely on the Arduino clock and it is the only one controlling the experiment (Controller). In the first option, the Raspberry Pi sends the stimulation values over the serial port by using the functions *Blink_from_Pi_serial* and *Custom_intensity_from_Pi_serial*. Using this method adds more than a hundred milliseconds of latency from when the data is sent from the Raspberry Pi until the lights are changing its physical state but because the Raspberry Pi is the only device that is taking decisions this delay will not be drifting. In the second option, either GPIO pin 21 or 20 on the Raspberry Pi is connected to the I^2^C adapter board (Fig. 23b). The I^2^C adapter board allows the connection of the mentioned Pi GPIO pins with the Arduino Digital Pins 20 and 21. These Arduino digital pins are usable for interrupt service routines (ISR) which will interrupt the main program and they will be executed when their logic state is changed externally by the Raspberry Pi GPIO pins. The time required to change the state of an Arduino output digital pin triggered by a Raspberry Pi GPIO pin 21 connected to Arduino digital pin 21 is 16 µs as measured with a Siglent SDS1104X-U Oscilloscope (Fig. 23c). However, the same process but actuating both matrices, which requires addressing all the matrix LEDs, increases the response time to 77 ms (Fig. 23d). Future customizations of the device could be designing matrices without individually addressable LEDs and fixed wavelengths which could be respond at µs scale. Using interrupts would be the way with less latency to trigger externally the Arduino from the Raspberry Pi keeping the later as controller and not relying on the Arduino Mega clock for stimulation intervals. The Arduino sketch using interrupts can be found in the repository as *OptoPi_ISR.ino and the Python script as record_video_interrupts_ISR.py*.

### 7.5 Experimental characterisation of the optogenetic modules

Here we validate experimentally the optogenetic capabilities of the OptoPi by using two stereotypical behaviours of the *Drosophila melanogaster* larvae: rolling and stopping.

Rolling is a nocifensive behaviour displayed by larvae when their nociceptive cells are being stimulated either mechanically or thermally. It is possible to elicit this behaviour without the stimuli that naturally trigger it by optogenetically activating either the sensory neurons, or downstream neurons of the nociceptive circuit. Here, we employed a driver line, *GMR72F11-Gal4,* which is expressed in interneurons of the nociceptive circuit and whose optogenetic activation has been shown to elicit robust rolling behaviour[13]. To activate these neurons we crossed this Gal4 to a UAS line encoding the red light activated ion channel CsChrimson (*UAS-CsChrimson*, BL79599)[7].

Stopping is a natural behaviour that larvae perform when they encounter an aversive stimulus, or during regular navigation when re-evaluating their direction of travel. However, stopping can also be elicited artificially by simultaneously inactivating all motorneurons, thus rendering the animals immobile. We employed this later approach by targeting all motorneurons (and a few other interneurons) using the vGlut-Gal4 (BL26160) driver line [14], [15]. We crossed this line to UAS-GtACR1, which encodes a green-light sensitive chloride channel, which upon activation by light inhibits the neurons where is being expressed[14].

**Fig. 24:**
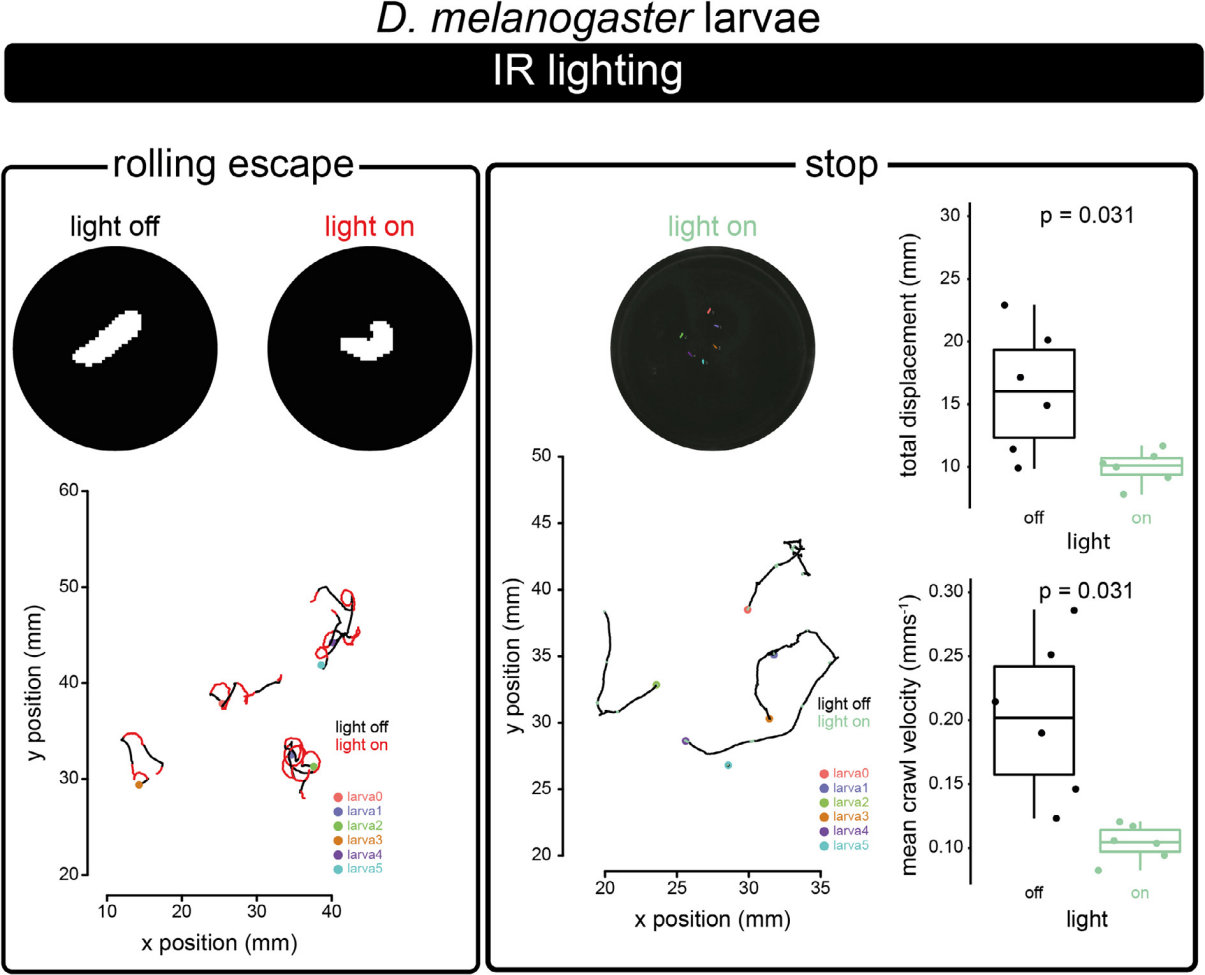
Experimental characterisation of the optogenetic module. Example *D. melanogaster* larvae optogenetic experiments. Left: Rolling escape behaviour elicited by exposing *GMR72F11-Gal4/UAS-CsChrimson* larvae to red light. Upper panels, example larval contours without and with red light exposure. Lower panel, tracks of larvae in a plate as they are exposed to dark and red light. Red portions of trajectory occur during exposure to red light. Right: Stopping behaviour elicited by exposing *VGlut-Gal4/UAS-GtACR1* larvae to pulses of green light. Upper panel left, still of larvae when exposed to green light. Upper panel right, total larval displacement during periods of time when green light is off vs on. Significance determined by paired Wilcoxon test performed using R package ggpubr. Lower panel left, green portions of trajectory occurring during exposure to green light. Lower panel right, mean crawl velocity of larvae during periods of time when green light is off vs on. Significance determined by paired Wilcoxon test performed using R package ggpubr.

For either experiment, 30 male and female adults were placed in cages placed on top of a grape juice agarose plate (3% agar, 25% grape juice) for 4 h. The plates were smeared with yeast paste containing 1 mM all-trans retinol (Sigma Aldrich, CAS116-31–4). Larvae for optogenetic experiments were collected 48 h following adult introduction to cages. *Drosophila* crosses were reared at 25C and in the dark. The experimental larvae were collected from the grape juice agarose plates using forceps, cleaned with water, and placed over a fresh agarose plate (1%). During behavioural assays, larvae were imaged using the side IR illumination and were kept in the dark, while intermittently exposed to red/green light via the blinking (20 s on 20 s off) protocol in OptoPi.ino. Thus, during periods when the larvae were in the dark, they could behave normally while exposure to red/green light optogenetically activated target neurons. *GMR72F11-Gal4/UAS-CsChrimson* larvae exhibited rolling behaviour only during red light stimulation and *VGlut-Gal4/UAS-GtACR1* larvae paused in response to green light stimulation (Fig. 1, Table 1, Supplementary Videos 22–23). Periods of exposure to red/green light are exported into a separate.csv file or can be directly annotated onto each video frame using the PiCamera module (e.g. where 1 might signify a light pulse and 0 might signify no light pulse). These behaviours can also be easily observed by colour coding their tracks dependent on whether they were being exposed to red/green light or in the dark (Fig. 24).

**Table 1:** Example settings for OptoPi that can be used to reproduce the example experiments of the figure. These combine settings determined by OptoPi.ino and user defined settings. Distance bottom (Db), Distance Top (Dt), IR illumination angle (IR), RGB angle (RGB), Camera distance (Cd), RGB brightness (RGB Br).

Experiment	Db	Dt	IR	RGB	Cd	IR Br	IR Lens	RGB Lighting	RGB Br	RGB lighting protocol
A – *D.*Fig. 21a D*.mel* adult offline ambient	2	7	45	180	138	100	off	255,255,255	80	constant
Fig. 21a D*.mel* adult online ambient	2	7	45	180	138	100	off	255, 255, 255	80	constant
Fig. 21a D*. mel* adult offline IR	1.4	3	66	142	69	100	on	off	0	constant
Fig. 21b D*. mel* 1st instar larvae offline IR	3.8	7	98	180	0	100	on	off	0	constant
Fig. 21c D*. rerio* offline ambient	4	7	98	180	0	100	off	255, 255, 255	80	constant
Fig. 24*(left)D. mel* larvae GMR72F11/CsChrimson offline IR	4	7	98	180	70	100	on	255,0,0	80	blinking (20 s on, 20 s off)
Fig. 24*(right) D. mel* larvae VGlut/GtACR1 offline IR	3.8	7	98	180	70	100	on	82, 255, 0	80	blinking (20 s on, 20 s off)

We therefore demonstrate that the OptoPi reliably elicits well-characterised optogenetically induced behaviours.

## Declaration of Competing Interest

The authors declare that they have no known competing financial interests or personal relationships that could have appeared to influence the work reported in this paper.
